# Nutrient and Amino Acid Digestibility of Fermented Cottonseed Meal and Fermented Cottonseed Protein Produced with Different Microbial Combinations in Growing Pigs

**DOI:** 10.3390/ani16213427

**Published:** 2026-10-30

**Authors:** Can Zhang, Wenqing Lu, Juntao Li

**Affiliations:** National Feed Engineering Technology Research Center, College of Animal Science and Technology, China Agricultural University, No. 2 Yuanmingyuan West Road, Beijing 100193, China; b20233040380@cau.edu.cn (C.Z.);

**Keywords:** fermented cottonseed meal, fermented cottonseed protein, nutrient digestibility, available energy, amino acid digestibility, prediction model, growing pigs

## Abstract

High-fiber unbalanced amino acid (AA) profiles and toxic free gossypol restrict cottonseed by-products for swine diets, while solid-state microbial fermentation can upgrade their nutrition. We prepared 103 fermented batches with diverse microbes under aerobic or anaerobic conditions, selected 10 representative groups via multivariate statistics for scaled-up fermentation and chemical analysis, and conducted two Youden square pig trials to determine available energy and standardized ileal digestible (SID) AAs before establishing relevant prediction equations. The metabolism experiment used 24.10 kg pigs to detect total tract digestibility and energy values, and an ileal cannula trial on 60.88 kg barrows adopted a nitrogen-free diet to correct endogenous amino acid losses. Fermentation largely lowered free gossypol, and short-term aerobic Aspergillus niger fermentation yielded the highest digestibility of most nutrients and essential amino acids. Digestible and metabolizable energy was positively related to crude protein (CP) and ash but was negatively linked to fiber fractions. SID lysine was negatively correlated with CP and ash yet was positively associated with fiber, while SID methionine and tryptophan displayed identical positive correlations with CP and negative correlations with fiber and free gossypol. Stepwise regression models (R^2^ = 0.57–0.93) allow rapid nutritional assessment via routine chemical analysis to guide precise pig feed production.

## Simple Summary

Cottonseed leftover after oil extraction is an inexpensive plant protein source for pig feed, yet harmful natural toxins and coarse fiber severely restrict its widespread feeding application. This research aimed to identify ideal microbial fermentation techniques to upgrade cottonseed nutritional quality and develop convenient evaluation methods that avoid costly pig feeding experiments. We prepared over one hundred fermented cottonseed batches with various microbe combinations, selected ten representative fermentation schemes, and then conducted digestion metabolism tests and ileal cannula trials on growing pigs to detect the usable energy and digestible amino acids of fermented cottonseed meal and fermented cottonseed protein. Results showed that short-term oxygen-rich fermentation with black mold achieved the best detoxification and nutrient absorption effects. We further constructed mathematical prediction formulas relying on regular feed chemical tests to rapidly calculate feed energy and digestible amino acid levels. This work provides straightforward production guidance for feed manufacturers to utilize cotton by-products as low-cost pig feed and cuts expenses from animal digestion trials.

## 1. Introduction

China ranks first globally in pig production and pork consumption [1]. In 2023, the total number of slaughtered commercial pigs reached 726.62 million head, with a total pork production of 57.94 million tons [2]. Rising pork demand has greatly increased the consumption of high-protein feedstuffs, but the competition for grain resources between human food and livestock production has intensified [3]. Cotton is a major oilseed crop grown widely in temperate and tropical regions, producing large quantities of processing by-products annually [4]. China produces approximately 10 million tons of cottonseed per year, with output continuing to rise [5,6]. After oil extraction, cottonseed yields cottonseed meal (CSM), hulls, linters, and crude oil [7].

The crude protein (CP) content within CSM varies from 40.67% to 59.67% on a feed basis [8]. However, its nutritional limitations restrict its use in swine diets: an unbalanced amino acid (AA) profile—deficient in sulfur-containing AAs (methionine and cysteine), lysine, and tryptophan—and the presence of free gossypol, a toxic anti-nutritional factor [9,10]. If gossypol can be reduced to safe levels, CSM would become an economical alternative plant protein source. In contrast, cottonseed protein (CSP) is a more refined product obtained through additional dehulling, low-temperature oil extraction, and detoxification processes, resulting in a higher protein concentration (typically 50–60% or more) and significantly lower fiber and free gossypol content compared to CSM. Nevertheless, CSP still suffers from AA imbalances similar to those of CSM.

Microbial solid-state fermentation has been shown to simultaneously degrade gossypol and generate bioactive metabolites, improving the feeding value of fermented CSM (FCSM) and fermented cottonseed protein (FCSP) as potential substitutes for corn and soybean meal [11]. Although fermented cottonseed products are used in ruminants, poultry, and aquaculture, research in growing pigs remains limited.

Given the substantial compositional differences between CSM and CSP—particularly in protein, fiber, and gossypol levels—their nutritional values for pigs cannot be assumed to be identical. Moreover, fermentation further modifies their nutrient profiles depending on the microbial strains and processing conditions, making a single fixed nutritional value inadequate for precision feeding. To address this, this study produced 103 batches of fermented cottonseed co-products using various microbial combinations. Ten representative samples were selected via multivariate analysis and further evaluated in digestion trials with growing pigs. The objectives were to assess nutrient and AA digestibility and to develop regression equations for predicting DE, ME, and SID AAs based on conventional chemical indicators, thereby providing a rapid evaluation tool for efficient application in pig production.

## 2. Materials and Methods

All animal procedures were approved by the Institutional Animal Care and Use Committee of China Agricultural University (Beijing, China; AW70506202-1-06) and performed at the Experimental Animal Center of China Agricultural University.

### 2.1. Cottonseed Meal Samples

CSM and CSP samples with different CP contents were obtained from commercial suppliers across various regions in China: C1, Xinjiang Herun Jinlan Biotechnology Co., Ltd. (Urumqi City, Xinjiang Uygur Autonomous Region, China); C2, Xinjiang Tekun Group Co., Ltd. (Urumqi City, Xinjiang Uygur Autonomous Region, China); C3, Xinjiang Tianzemu Plant Protein Technology Co., Ltd. (Urumqi City, Xinjiang Uygur Autonomous Region, China); C4, Xinjiang Hipro Biotechnology Co., Ltd. (Urumqi City, Xinjiang Uygur Autonomous Region, China); C5, Lingshou County Lvde Agricultural and Sideline Products Sales Department (Shijiazhuang City, Hebei Province, China). The analyzed nutrient components of each cottonseed meal were presented in Table 1.

### 2.2. Fermented Cottonseed Meal and Fermented Cottonseed Protein Samples

Exp. 1: The fermentation substrates, cottonseed protein (samples C1–C3) and cottonseed meal (samples C4–C5), were ground to pass through 80-mesh (0.18 mm) and 16-mesh (1.18 mm) sieves, respectively. All substrates were sterilized by autoclaving at 121 °C for 20 min prior to fermentation to eliminate background microbial contamination. All microbial strains and neutral protease were obtained as commercial powders with the following specific titers: Aspergillus oryzae (1.5 × 10^10^ CFU/g), Aspergillus niger (1.5 × 10^10^ CFU/g), Saccharomyces cerevisiae (3.0 × 10^10^ CFU/g), Bacillus subtilis (2.0 × 10^11^ CFU/g), Bacillus coagulans (1.0 × 10^10^ CFU/g), Lactobacillus plantarum (1.0 × 10^11^ CFU/g), and neutral protease (3.3 × 10^5^ U/g). The strains were obtained from commercial suppliers (the specific strain designations and sources are listed in Table S1 in the Supplementary Materials). For inoculation, each powdered strain was first suspended in sterile distilled water to form a uniform suspension, which was then sprayed evenly onto the substrates with thorough mixing to ensure homogeneous distribution, at the inoculation amounts specified in Table 2 (aerobic fermentation) and 3 (anaerobic fermentation).

For aerobic fermentations, after mixing the inoculum with the substrate, the mixture was placed in sterile aerobic fermentation boxes (polypropylene containers with breathable lids, dimensions: 28 × 18.5 × 9.3 cm) and incubated statically in a constant-temperature incubator at the temperatures and times specified in Table 2 and Table 3. For anaerobic fermentations, the inoculated substrate was placed in sterile anaerobic fermentation bags (sealed, dimensions: 23 × 30 cm, with oxygen-absorbing sachets) and incubated statically in a constant-temperature incubator for 72 h. Both the fermentation boxes and bags were sterilized by autoclaving prior to use. After fermentation, all products were dried at 45 °C in a forced-air drying oven until the moisture content reached approximately 10%, then ground and stored in sealed containers at 4 °C until further analysis.

The fermentation parameters for aerobic and anaerobic fermentation are summarized in Table 2 and Table 3, respectively. A total of 103 batches of FCSM and FCSP were prepared using different fermentation protocols, with each fermentation treatment performed in triplicate. Conventional nutritional components of all fermented samples were determined, and the corresponding analytical results were presented in Table 4.

Nutrient data (crude protein, peptide content, and protein solubility) from the 103 fermented batches were preprocessed and standardized using Z-score normalization to eliminate scale effects. The standardized data were then subjected to principal component analysis (PCA) for dimensionality reduction; principal components with eigenvalues > 1, cumulatively explaining > 80% of the total variance, were retained. K-means clustering was performed on the PCA scores, and the optimal number of clusters (k = 10) was determined using the elbow method and silhouette coefficient. Within each cluster, the Euclidean distance from each sample to its respective cluster centroid was calculated based on the standardized variables. From each cluster, the sample with the largest distance from the cluster centroid (i.e., the most extreme sample within that cluster) was selected as a representative to capture the maximum variation within each cluster while ensuring overall diversity coverage. The selected samples were further validated by independent sample *t*-tests to confirm significant differences (*p* < 0.05) in at least one of the three key indicators compared with the remaining samples. Based on the above multivariate analyses, ten representative fermentation treatments (F1–F10) (Table 5) were finally screened out. Subsequently, scale-up fermentation experiments (50 kg raw substrate per batch) were implemented in accordance with the optimized fermentation procedures to reproduce the 10 screened FCSMs and FCSPs (Table 6).

### 2.3. Animals, Diets, and Experimental Design

Exp. 2: Eleven crossbred growing barrows (Duroc × Landrace × Yorkshire; initial body weight: 24.10 ± 1.98 kg) were randomly allocated to 11 dietary treatments. A replicated 11 × 6 Youden square design (11 diets × 6 experimental periods) was adopted in this trial. Each experimental period lasted 10 days, consisting of a 7-day dietary adaptation period and a 3-day total collection period for feces and urine. The 11 experimental diets consisted of one basal diet and 10 test diets, in which 25% of the energy-yielding ingredients of the basal diet were replaced by FCSM or FCSP. The daily feed supply was restricted to 4% of individual pig body weight [12], with equal portions provided at 08:00 and 16:00 daily.

The ingredient composition and analyzed nutrient contents of all experimental diets (Table 7) were formulated to meet or exceed the nutrient requirements of growing pigs as recommended by the NRC (2012) [13]. All pigs were individually housed in metabolism crates (1.4 × 0.45 × 0.6 m) fitted with self-feeders, nipple drinkers, and slatted floors. Sieve baffles and independent urine collection trays were mounted under the slatted floors to achieve complete separate collection of feces and urine. The ambient room temperature was maintained at 20–24 °C, and all pigs had ad libitum access to drinking water throughout the trial.

Exp. 3: Twenty-two Duroc × Landrace × Yorkshire crossbred barrows with an initial body weight of 60.88 ± 3.00 kg were surgically fitted with simple T-cannulas at the distal ileum, according to the procedure described by Stein et al. [14]. After surgery, all pigs were allowed a 7-day postoperative recovery period during which they were fed a standard diet and monitored daily for feed intake, general health, and wound healing. Following recovery, all cannulated pigs were randomly allocated to 11 dietary treatments in a replicated 11-treatment × 3-period Youden square experimental design. Each experimental cycle consisted of a 5-day diet adaptation period and a 2-day ileal digesta collection period.

The experimental diets comprised 10 test diets and one nitrogen-free diet. Specifically, FCSM or FCSP served as the primary amino acid (AA) sources in the 10 test diets, while the nitrogen-free diet was used to quantify the basal endogenous losses of CP and AAs. Chromic oxide was incorporated into all diets at a proportion of 0.3% as an indigestible inert marker. Vitamin and mineral premixes were supplemented to satisfy or surpass the nutrient requirements of growing pigs outlined in the NRC (2012) [13]. The ingredient composition and analyzed nutrient profiles of the experimental diets for Experiment 3 are presented in Table 8 and Table 9, respectively.

The daily feed allowance was restricted to 4% of each pig’s body weight [12], which was equally divided into two meals and supplied at 08:00 and 16:00 daily. After adequate postoperative recovery from surgery, pigs were subjected to a 7-day experimental cycle, consisting of 5 days of dietary acclimation and 2 consecutive days of ileal digesta sampling. Daily digesta collection was performed for 9 h each day, starting at 08:00, in accordance with the sampling protocol reported by Stein et al. [14].

### 2.4. Sample Collection

In Exp. 2, total fecal excreta of each pig were collected at 08:30 on days 8 to 10 of each experimental period and immediately stored at −20 °C. Urine was separately collected into plastic buckets prefilled with 50 mL of 6 mol·L^−1^ hydrochloric acid to fix nitrogen. After collection, urine samples were filtered through cotton gauze, and 10% of the daily urine output was retained and stored at −20 °C. At the termination of Exp. 2, all daily fecal samples were thawed and thoroughly homogenized, and a 300 g subsample was reserved for subsequent chemical analysis. Daily urine aliquots were also thawed and blended, and two 50 mL subsamples were prepared for subsequent detection. All fecal subsamples were oven-dried at 65 °C for 72 h and then ground to pass through a 40-mesh sieve. The individual body weight of each pig was recorded at the initiation and termination of each experimental period, and daily feed supply was fully documented throughout the trial.

In Exp. 3, on days 6 and 7 of each digesta collection period, plastic sampling bags were fastened to the cannula outlet. Digesta-filled bags were replaced at least every 30 min and immediately stored at −20 °C to inhibit the microbial breakdown of amino acids (AAs) in digesta [15]. Upon completion of the trial, all digesta samples were thawed and homogenized by individual pig and experimental period, followed by subsampling and lyophilization using a vacuum freeze dryer (Tofflon Freezing Drying Systems, Shanghai, China). Pig body weights were measured at the start and end of each period, and daily feed provision was recorded. Pigs were fasted overnight after each experimental cycle before switching to the next dietary treatment on the following morning.

### 2.5. Chemical Analysis

All samples, including the initial cottonseed meal/cottonseed protein substrates (C1–C5), fermented products (F1–F10), experimental diets, feces, urine, and ileal digesta, were analyzed in duplicate. Dry matter (DM) and crude ash were quantified following AOAC (2012) official Methods 934.01 and 942.05, respectively [16]. CP was measured using the Kjeldahl procedure (AOAC Method 976.05), with nitrogen multiplied by 6.25 as the conversion factor [17]. Neutral detergent fiber (NDF) and acid detergent fiber (ADF) were determined in accordance with Van Soest et al. [18]. Free gossypol (FG) concentrations in the initial substrates (C1–C5) and fermented products (F1–F10) were determined according to the Chinese national standard GB/T 13086-2020 [19]. Chromium concentrations in the experimental diets and ileal digesta were quantified by atomic absorption spectrophotometry in line with AOAC (2006) guidelines [20]. The urine was filtered, and 4 mL of the filtrate was placed in a small iron pan lined with filter paper and dried in an oven at 65 °C for 8 h. Then, the gross energy (GE) of diets, feces, and urine was determined with an isoperibol bomb calorimeter (Parr 6400, Moline, IL, USA). Organic matter (OM) was calculated by the formula OM = DM − crude ash. Small peptides were determined following the National Standard GB/T 22492 [21], and protein solubility was determined following the AOCS Official Method [22].

Total AAs in the diets and ileal digesta were analyzed using an automatic amino acid analyzer (Hitachi L-8900, Tokyo, Japan) in accordance with the Chinese national standard GB/T 18246-2019 [23]. As tryptophan is susceptible to degradation during acid hydrolysis, it was separately determined by high-performance liquid chromatography (Agilent 1200 Series, Santa Clara, CA, USA) following alkaline hydrolysis pretreatment.

### 2.6. Calculations

(1) Apparent total tract digestibility (ATTD)

The ATTD of nutrients in experimental diets and test ingredients (FCSM or FCSP) was calculated using the substitution method according to Adeola [24]:

ATTD of dietary nutrients (%) = (Total nutrient intake − Total nutrient excretion in feces)/Total nutrient intake × 100%

ATTD of nutrients in fermented cottonseed meal (%) = (ATTD of test diet nutrient − (100% − X1%) × ATTD of the corresponding nutrient in the basal diet)/X1%

where X1% is the substitution ratio of the energy component in the basal diet;

(2) Digestible energy (DE) and metabolizable energy (ME) of diets

DE and ME contents of experimental diets were calculated according to the following equations [24]:

DE (MJ/kg, DM) = (GE intake, MJ − fecal energy, MJ)/DM intake, kg

ME (MJ/kg, DM) = (GE intake, MJ − fecal energy, MJ − urine energy, MJ)/DM intake, kg

DE and ME values of FCSM and FCSP.

DE and ME contents of FCSM and FCSP were calculated according to the following equations [25]:

DE (MJ/kg, DM) = [DE of the test diet − DE of the basal diet × (1 − X1%)]/X1%/X2%

ME (MJ/kg, DM) = [ME of the test diet − ME of the basal diet × (1 − X1%)]/X1%/X2%

where X1% is the substitution ratio of the energy component (corn) in the basal diet; X2% is the level of corn in the basal diet;

(3) Apparent ileal digestibility (AID) of amino acids

AID of AAs was calculated according to Stein et al. [26]:

AID of AAs (%) = 100% − (Diet chromium content, g/kg DM × Ileal digesta AA content, g/kg DM)/(Ileal digesta chromium content, g/kg DM × Diet AA content, g/kg DM) × 100%

Standardized ileal digestibility (SID) of amino acids.

SID of AAs was calculated based on the method of Stein et al. [26]:

Basal ileal endogenous AA losses (BIAA) (g/kg DMI) = Ileal digesta AA content, g/kg DM × Diet chromium content, g/kg DM/Ileal digesta chromium content, g/kg DM

SID (%) = AID, % + (BIAA, g/kg DMI/Test diet AA content, g/kg DM × 100%)

In all formulas above, DM and DMI represent dry matter and dry matter intake, respectively.

SID AA (g/kg DM) = test diet AA content, g/kg DM × (SID/100)

### 2.7. Statistical Analysis

All statistical analyses were performed using IBM SPSS Statistics 24.0 (IBM Corp., Armonk, NY, USA). In Exp. 1, nutrient datasets of 103 fermented cottonseed co-products were preprocessed and standardized. Subsequently, multivariate analyses including principal component analysis (PCA) for dimensionality reduction, cluster analysis, hierarchical distance classification and significance testing were implemented to select 10 representative fermented substrates for subsequent animal trials.

For Exp. 2 and Exp. 3, all data were subjected to analysis of variance (ANOVA) using SPSS via the MIXED procedure. Dietary treatment was treated as a fixed factor in the statistical mixed model, while individual pig and experimental period were included as random effects. Least-squares means were calculated for each treatment, and differences among treatments were compared using pairwise comparisons with Tukey adjustment for multiple comparisons. Furthermore, Pearson correlation analysis and stepwise multiple linear regression were performed to quantify relationships between routine chemical constituents and nutritional parameters, and to develop prediction equations for DE, ME, and SID AAs. Differences were regarded as significant at *p* < 0.05, and a tendency was considered at 0.05 ≤ *p* < 0.10.

## 3. Results

### 3.1. Effects of Different Fermentation Processes on the Conventional Nutritional Components of FCSM and FCSP

Compared with the corresponding unfermented substrates (C1, C2, C3, and C5), the free gossypol contents of all fermented treatments were significantly reduced (*p* < 0.01), with the only exception of F9 (Table 10, Table 11, Table 12 and Table 13). Although no significant difference in free gossypol concentration was observed between F9 and C2, F9 showed a numerical reduction in free gossypol. The OM content of F7, F10 (Table 13) and F9 (Table 11) was significantly lower than those of their corresponding unfermented substrates (*p* < 0.01). Similarly, the content of DM of F1 and F6 (Table 10), F3 and F9 (Table 11), and F8 (Table 12) was significantly lower than their respective unfermented substrates (*p* < 0.01). Compared with C1 (Table 10), F1, F5, and F6 exhibited a significant reduction in NDF content (*p* < 0.01), and F5 had a significantly decreased CP content (*p* < 0.01). Compared with C2 (Table 11), CP content was significantly increased in F3 but decreased in F9 (*p* < 0.01). F7 contained higher NDF and ADF levels than C5 (Table 13), while both F7 and F10 had significantly lower GE values (*p* < 0.05).

### 3.2. The ATTD of Nutrients and Available Energy in the Diets of Growing Pigs in Exp. 2

The ATTD of nutrients and available energy values of experimental diets were shown in Table 14. Dietary treatments significantly affected the ATTD of DM, OM, CP, ADF, GE, as well as the available values (DE and ME) (*p* < 0.05). In contrast, no significant differences were observed in the ATTD of neutral detergent fiber (NDF) and the ME/DE ratio across all dietary groups (*p* > 0.05). Compared with the basal diet, Test Diet 2 exhibited significantly lower ATTD of DM, OM, and GE (*p* < 0.05), whereas no significant differences were detected for the other test diets. Among different test diet groups, Test Diet 2 had significantly lower ATTD of DM, OM, and GE than Test Diets 1, 5, 6, and 9 (*p* < 0.05). Additionally, the CP ATTD of Test Diet 2 (80.37%) was significantly lower than those of Test Diets 6 and 9 (*p* < 0.05). For ADF ATTD, Test Diet 9 showed the highest value (70.51%), while Test Diets 2 (40.23%) and 4 (39.71%) had significantly lower ADF ATTD (*p* < 0.05).

In terms of energy availability, Test Diet 9 had the greatest DE content (17.00 MJ/kg DM), and Test Diet 6 had the highest ME content (16.33 MJ/kg DM). By comparison, Test Diet 2 yielded the lowest DE (14.89 MJ/kg DM) and ME (14.45 MJ/kg DM) values. The ME/DE ratio ranged from 92.84% to 96.99% across all treatments, with no significant differences observed among dietary groups.

### 3.3. The ATTD of Nutrients and Available Energy in the FCSM and FCSP of Growing Pigs in Exp. 2

The ATTD of nutrients and available energy values (DE and ME) of FCSM and FCSP samples were presented in Table 15. Significant differences were observed in the ATTD of DM, OM, CP, and GE, as well as in the DE content among different FCSM and FCSP samples (*p* < 0.05). Meanwhile, no significant differences were detected in the ME content and ME/DE ratio (*p* > 0.05). F2 exhibited significantly lower ATTD of DM, OM, and GE than that of F3, F5, F6, and F9 (*p* < 0.05). Meanwhile, F2 had the lowest ATTD of CP (64.72%), which was significantly lower than that of F3 and F6 (*p* < 0.05). The DE values of 10 FCSM and FCSP samples ranged from 12.58 to 21.42 MJ/kg DM, and the DE content of F2 was the lowest (12.58 MJ/kg DM), which was significantly lower than those of F3, F5, F6, and F9 (*p* < 0.05). The ME content of all test samples ranged from 11.90 to 19.31 MJ/kg DM, and the ME/DE ratio ranged from 82.96% to 95.82%.

### 3.4. The AID of Amino Acids of FCSM and FCSP of Growing Pigs in Exp. 3

As presented in Table 16, except for Trp and Pro, significant differences were observed in the apparent ileal digestibility (AID) of all amino acids among treatments (*p* < 0.05). For essential amino acids, Group 3 exhibited a significantly greater AID of leucine (Leu) and isoleucine (Ile) than Groups 1, 4, 5, 7, 9, and 10 (*p* < 0.01). The AID of His, Val, and Phe in Group 3 was significantly greater than that in Groups 1, 4, 5, 7, and 9 (*p* < 0.01). Excluding Group 2, Group 3 had a significantly higher AID of Thr than all other test groups. Group 1 had the lowest AID of Lys and Arg, whereas Group 8 exhibited the lowest AID of Met (*p* < 0.01).

For non-essential amino acids, Group 3 had a greater AID of Cys than Group 7 (*p* = 0.03). Excluding group 2, the AID of Ser and Asp in group 3 was higher than the remaining test groups (*p* < 0.01). Groups 2 and 3 had significantly higher AID of Ala and Glu than Groups 1, 4, 5, and 7 (*p* < 0.01). Furthermore, Group 3 had a greater AID of Gly than Groups 1, 4, 5, 7, and 9 (*p* < 0.01). In contrast, Group 7 had the lowest AID of Cys, and Groups 1, 2, and 5 exhibited lower AID of Tyr (*p* < 0.01).

### 3.5. The SID of AAs of FCSM and FCSP of Growing Pigs in Exp. 3

As shown in Table 17, except for Cys, Met, Trp, and Pro, the SID of the remaining AAs differed significantly among test groups.

For essential AAs, Groups 2 and 3 had a significantly greater SID of Ile than Groups 1, 4, and 5. Group 3 exhibited a significantly higher SID of Lys than Groups 1, 4, 5, 6, 7, and 9. Specifically, Group 3 had a greater SID of Leu and Arg than Groups 1, 4, 5, and 7, a higher SID of His and Phe than Groups 1, 4, 5, 7, and 9, and a greater SID of Val than Groups 1, 4, and 5. Excluding Groups 2 and 8, the SID of Thr in all other groups was significantly lower than that in Group 3.

For non-essential amino acids, Group 3 showed significantly higher SID of Ser than all test groups, except Groups 2 and 8. Meanwhile, Group 3 had greater SID of Ala than Groups 1, 4, 5, and 7, higher SID of Asp than Groups 1, 4, 5, 7, and 9, and higher SID of Glu than Groups 1, 4, and 5. Additionally, Groups 1 and 5 had relatively low SID of Tyr, and Group 1 presented the lowest SID of Gly among all experimental treatments.

### 3.6. Correlation Analysis of Routine Chemical Composition and Available Energy Values of Ten FCSM and FCSP Samples Fed to Growing Pigs

The correlation analysis between available energy values and routine chemical compositions was shown in Table 18. DE exhibited an extremely significant positive correlation with crude protein (CP, r = 0.80, *p* < 0.01) and a significant positive correlation with crude ash (Ash, r = 0.74, *p* < 0.05). Meanwhile, DE was negatively correlated with multiple nutritional indicators. Extremely significant negative correlations were observed with neutral detergent fiber (NDF, r = −0.82, *p* < 0.01) and acid detergent fiber (ADF, r = −0.81, *p* < 0.01), while significant negative correlations existed with organic matter (OM, r = −0.74, *p* < 0.05) and dry matter (DM, r = −0.69, *p* < 0.05). ME had an extremely significant positive correlation with DE (r = 0.96, *p* < 0.01) and a significant positive correlation with CP (r = 0.70, *p* < 0.05). In addition, ME showed significant negative correlations with NDF (r = −0.72, *p* < 0.05), ADF (r = −0.71, *p* < 0.05) and DM (r = −0.64, *p* < 0.05).

For the SID of amino acids, the correlation analysis with routine chemical compositions and free gossypol was presented in Table 19. SID Lys was significantly negatively correlated with crude ash (Ash, r = −0.31) and crude protein (CP, r = −0.39), while being positively correlated with organic matter (OM), neutral detergent fiber (NDF), and acid detergent fiber (ADF) (*p* < 0.05). SID Trp displayed extremely significant positive correlations with Ash and CP, alongside extremely significant negative correlations with NDF, ADF, and free gossypol (*p* < 0.01). SID Met showed extremely significant positive correlations with Ash, CP, and SID Trp, and extremely significant negative correlations with NDF, ADF, and free gossypol (*p* < 0.01). SID Thr had no significant linear correlation with routine chemical compositions and free gossypol (*p* > 0.05).

Based on the correlation analysis, prediction equations for DE, ME, and SID of amino acids in FCSM and FCSP for growing pigs were developed. The optimal regression equations, as listed in Table 20, were presented as follows:

DE (MJ/kg DM) = 20.96 − 5.52 Ash + 1.02 CP + 0.56 ADF − 0.23 DM (*p* < 0.05, R^2^ = 0.81)

ME (MJ/kg DM) = 2.13 − 0.42 Ash + 0.02 DM + 0.91 DE (*p* < 0.01, R^2^ = 0.93)

SID Lys (%) = 298.32 − 1.37 DM − 4.50 Ash − 0.50 OM + 1.26 NDF − 3.73 GE − 0.08 free gossypol (*p* < 0.01, R^2^ = 0.71)

SID Met (%) = −2.1 − 4.45 Ash − 0.05 OM + 0.81 CP + 0.73 ADF − 0.01 free gossypol (*p* < 0.01, R^2^ = 0.76)

SID Thr (%) = 156.92 − 0.77 DM − 0.36 OM + 0.80 NDF − 2.22 GE − 0.04 free gossypol (*p* < 0.01, R^2^ = 0.57)

SID Trp (%) = −16.01 + 0.06 DM- 2.25 Ash + 0.55 CP + 0.20 NDF (*p* < 0.01, R^2^ = 0.78).

## 4. Discussion

### 4.1. Effects of Different Fermentation Processes on Nutritional Components of FCSM and FCSP

Soybean meal (SBM) is the most widely used protein ingredient in commercial pig feed [27]. However, its continuous cost increases and price volatility have driven the exploration of feasible alternative plant protein resources. CSM and cottonseed protein, which contain abundant salt-soluble, water-soluble, and alkali-soluble proteins, are considered promising protein supplements for swine diets [28,29]. However, the wide application of raw CSM is severely restricted by its high fibrous fractions, imbalanced amino acid profiles, and toxic gossypol, which adversely impair nutrient digestibility and animal growth performance. Microbial fermentation combined with exogenous enzyme supplementation is an effective strategy to optimize the nutritional matrix of CSM, which simultaneously degrades anti-nutritional factors and structural fiber and remodels the distribution of carbon and nitrogen pools [30,31].

A total of 103 fermented CSM samples were preliminarily analyzed for nutritional composition. After preprocessing, multivariate statistical analyses, including principal component analysis, cluster classification, and significance screening, were performed to screen 10 representative fermentation treatments with distinct strain combinations, oxygen regimes, and fermentation durations for subsequent 50 kg scaled-up fermentation trials. Previous studies have reported that conventional unfermented cottonseed co-products contain 36.0–55.0% CP, 11.67–41.52% NDF, and 4.43–29.26% ADF, with a GE ranging from 14.53 to 18.11 MJ/kg [32,33,34]. In the present experiment, the CP of fermented substrates varied from 46.30% to 63.57%, NDF from 9.08% to 34.10%, and ADF from 4.47% to 20.00%, which fell within the reported range of modified cottonseed products. Most nutritional indices presented coefficients of variation greater than 10%, which provided sufficient nutritional variation for evaluating fermentation modification effects and establishing accurate nutrient and energy prediction equations.

For the aerobic fermentation series of C1 substrates (F1 and F5), the pronounced reductions in NDF and ADF potentially confirmed the massive hydrolysis of cellulose and hemicellulose to soluble monosaccharides. Although OM content remained relatively stable, DM decreased significantly, indicating altered carbon flow under aerobic monoculture fermentation. The soluble carbon released from fiber degradation was not completely oxidized and dissipated as CO_2_. Instead, yeast and Bacillus strains may have diverted most carbon skeletons for microbial single-cell protein synthesis, replenishing organic nitrogen fractions and partly offsetting respiratory carbon loss [35]. This biomass retention mechanism dominated short-term aerobic fermentation (24–48 h). Specifically, F1 (*Saccharomyces cerevisiae* fermentation) significantly increased the CP content from 61.68% to 62.82% and reduced NDF by 51.2%, which was attributed to the synergistic effects of yeast cellulase hydrolysis and microbial nitrogen anabolism [36]. In contrast, F5 (single *Bacillus subtilis* aerobic fermentation) exhibited a notable CP decline despite identical fiber degradation. The excessive secretion of protease by Bacillus subtilis triggered an intensive hydrolysis of intrinsic cottonseed protein, and the decomposed free amino acids were preferentially utilized for respiratory energy provision rather than microbial biomass synthesis. Simultaneously, partial nitrogen was lost in the form of volatile ammonia, ultimately resulting in net nitrogen loss and reduced CP content [37].

Long-term anaerobic compound fermentation (72 h) induced distinct carbon mineralization. For F6 and F9 treated with compound microbial strains and neutral protease, synchronous decreases in DM and OM were observed. Exogenous protease disrupted protein cross-linking in the cell wall matrix, released encapsulated fibrous carbohydrates, and improved the substrate accessibility of anaerobic microbiota [38]. Diverse anaerobic microorganisms sequentially conducted hydrolysis, acidification, and methanogenesis, which may lead to substantial organic carbon loss via CO_2_ and CH_4_ emission. The inefficient conversion of organic carbon to microbial biomass further caused a negative total solid mass balance [33]. Consistent with this, F9 (C2 substrate) exhibited the greatest OM reduction (from 91.63% to 79.89%), accompanied by significant CP degradation (from 59.91% to 51.48%) and decreased GE values. These results demonstrated that prolonged multi-strain anaerobic fermentation induces excessive carbon and nitrogen mineralization, thereby deteriorating the nutritional value of fermented products [39].

Not all anaerobic fermentation caused pronounced OM depletion. The C5 lactic acid bacteria-dominated anaerobic treatments (F7, F10) displayed a unique pattern. OM declined significantly, while DM remained unchanged. Lactic acid bacteria lack complete cellulolytic enzyme systems and only utilize soluble free sugars without degrading lignocellulose [28]. Minor soluble carbon was released as CO_2_ and volatile fatty acids, reducing the relative proportion of OM. Undegraded fiber and mineral components were passively concentrated, raising NDF and ADF contents without altering the total dry solid content [40]. For the C3 group (F2, F4, F8), no significant shifts in OM, CP, NDF, ADF, or GE were observed, indicating the C3 substrate contained limited readily fermentable carbon sources to sustain vigorous microbial catabolism, resulting in weak modification effects regardless of aerobic or anaerobic inoculation.

Fiber degradation capacity was strictly strain-dependent and decoupled from oxygen conditions. Single aerobic fungi or Bacillus possessed complete cellulase, xylanase, and pectinase cascades. Hence, F1, F5, and F6 (C1) achieved remarkable NDF and ADF degradation [41]. The C2 inherently contained lower baseline fiber contents, and neither aerobic F3 nor anaerobic F9 generated sufficient fibrolytic enzymes to further reduce NDF. Lactic acid bacteria lacked fiber-degrading enzymes entirely, so F7 and F10 exhibited elevated NDF and ADF due to the passive content of recalcitrant fiber after soluble carbohydrate consumption [40].

Variations in gross energy directly reflected the quantity of residual fermentable organic carbon. F1 had slightly elevated GE, as fibrous polysaccharides were hydrolyzed into readily digestible small-molecule oligosaccharides that carry higher energy density than intact structural fiber [42]. By contrast, long-term anaerobic F9, F7, and F10 showed significant GE reduction, owing to sustained gaseous carbon loss over 72 h fermentation [43]. No GE differences were detected in the C3 series, consistent with its limited fermentable carbon pool and weak microbial metabolic intensity.

Free gossypol, the core anti-nutritional indicator, was significantly decreased across all fermented groups, except F9, relative to their respective raw substrates. Microbial oxidases and reductive enzymes break down the toxic polyphenol structure of free gossypol, and longer fermentation time generally strengthens detoxification efficiency [38]. However, F9 suffered severe OM and CP loss, indicating that overlong anaerobic fermentation achieves gossypol removal at the cost of substantial nutritional degradation.

Collectively, oxygen environment, strain combination, and fermentation duration jointly regulate carbon–nitrogen partitioning, fiber degradation, and anti-nutrient detoxification [44]. Short-term (24–48 h) aerobic fermentation using single yeast or Aspergillus strains represented the optimal modification strategy. These systems balanced fiber hydrolysis, microbial protein anabolism, and free gossypol degradation, avoiding the excessive carbon/nitrogen mineralization observed in 72 h composite anaerobic fermentation. Lactic acid bacteria-only anaerobic fermentation merely realized partial detoxification without improving fibrous or protein nutritional quality, while raw substrates with low readily fermentable carbon (C3) exhibited weak fermentation modification potential, regardless of processing regimes. From industrial application perspectives, the fermentation conditions evaluated in this study (30–37 °C, 24–48 h aerobic mono-strain fermentation) show promise for producing high-quality fermented cottonseed meal. However, further validation through large-scale production trials, economic analysis, and comprehensive safety assessments, including mycotoxin screening, is required before these conditions can be conclusively recommended for commercial application.

### 4.2. Effects of FCSM and FCSP on ATTD of Nutrients in Growing Pigs

The methods for determining the available energy value of feeds include the direct method, substitution method, and prediction equation method [27]. CSM is not suitable for the direct method due to its palatability issues and potential mycotoxin contamination. The FCSM and FCSP obtained through fermentation may contain mycotoxins. Meanwhile, the prediction equation for the available energy of FCSM and FCSP in growing pigs has not yet been established, so the substitution method is an appropriate approach to determine their available energy value. The substitution method requires replacing the energy-supplying components in the basal diet with a certain proportion of FCSM and FCSP. To reduce the interaction effects between different nutrients, it is necessary to ensure that the proportions of corn and soybean meal in both the basal diet and the experimental diets remain consistent. To obtain relatively accurate available energy values using the substitution method, a suitable substitution proportion must be determined [27]. In theory, the higher the substitution ratio of a test ingredient, the closer the measured available energy value is to the true value [45]. Villamide [45] and Feng [46] reported that available energy values obtained with an ingredient substitution ratio above 20% are relatively accurate. However, an excessively high inclusion rate may lead to considerable differences in nutritional composition between the basal diet and the experimental diets, resulting in unbalanced nutrient contents across dietary treatments and thus compromising the evaluation of available energy values of the feed ingredient [47]. To determine accurate available energy values of FCSM and FCSP by the substitution method, 25% of the energy-providing ingredients in the basal diet were replaced with the corresponding fermented feedstuffs in this study. Except for Diet 2, there were no significant differences in the ATTD of nutrients in growing pigs compared with the basal diet; the ATTD of CP was numerically higher in all other treatment groups than in the basal diet group. These results indicated that replacing 25% of the energy-supplying components in the basal diet did not exert adverse effects on growing pigs. A study showed that, under amino acid balance, CSM can fully replace soybean meal without compromising growth and health while improving nutrient digestibility [48]. Gu et al. found that the FCSM diet, formulated by substituting 6% SBM with FCSM, showed significantly greater digestibility of DM, OM, CP, GE, and AAs compared with the SBM diet [49]. Our results were in agreement with the above study. Studies have demonstrated that the CSM effectively reduces free gossypol content [50] and degrades macromolecular proteins into small peptides. Oligopeptides with low molecular weight can be rapidly absorbed via the intestinal mucosa, thereby improving the nutrient utilization efficiency of the diet [51]. Comparison among test groups showed that the ATTD of DM, OM, and GE of diets and ingredients in the test 2 group were significantly lower than those in the test 5, 6, and 9 groups. The ATTD of CP of diets and ingredients in the test 2 group was significantly decreased relative to the test 6 group. Moreover, the ATTD of ADF in diets in the test 2 and 4 groups was significantly lower than that in the test 9 group. Consequently, it can be concluded that the test 6 and 9 groups possessed favorable ATTD of nutrients.

### 4.3. Effects of FCSM and FCSP on AID and SID of Nutrients in Growing Pigs

Experimental Group 3, which was fermented with *Aspergillus niger*, consistently enhanced the AID of the majority of essential and non-essential amino acids. In contrast, Groups 1, 4, 5, and 7 showed significantly lower digestibility values across both AID and SID measurements. Zhang et al. indicated that *A. niger ZD-8* demonstrated the greatest efficacy in improving crude protein content, total amino acids, essential amino acids, and their respective in vitro digestibility, outperforming the other tested microorganisms among the six fungal strains evaluated (*C. capsuligena ZD-1*, *C. tropicalis ZD-3*, *S. cerevisiae ZD-5*, *A. terricola ZD-6*, *A. oryzae ZD-7*, and *A. niger ZD-8*) [51]. Specifically, fermentation with *A. niger ZD-8* significantly increased the levels of methionine, lysine, and threonine, as well as their digestibility [52]. Our results were consistent with the above findings. During solid-state fermentation, *A. niger* secretes substantial amounts of extracellular proteases, particularly acid proteases, which effectively degrade the macromolecular storage proteins of cottonseed meal into smaller peptides and free amino acids, thereby facilitating intestinal absorption and reducing endogenous losses [53]. In contrast, Groups 1, 4, 5, and 7 consistently ranked among the lowest for both AID and SID across multiple AAs. This pattern may be attributed to differences in the fermentation substrate, microbial strains, duration, or temperature regimes among these experimental groups. Inadequate fermentation could fail to effectively degrade the cell wall matrix and protein–polysaccharide complexes, thereby limiting access of digestive enzymes to peptide bonds and reducing AA release at the terminal ileum [54].

Among the groups with inferior amino acid digestibility, Group 7 is of particular interest for discussion. Group 7 employed a mixed fermentation of *Bacillus coagulans* and *Lactobacillus plantarum* yet consistently exhibited the lowest AID of Cys and significantly lower SID values for multiple essential amino acids compared with Group 3 (*A. niger fermentation*). Unlike *A. niger*, which secretes substantial amounts of extracellular acid proteases capable of extensively degrading macromolecular storage proteins, *B. coagulans* and *L. plantarum* are primarily known for their lactic acid-producing capabilities and oxygen consumption rather than potent proteolytic activity [55,56]. Consequently, the macromolecular proteins in cottonseed meal may have remained largely intact in Group 7, limiting the release of absorbable peptides and free amino acids in the small intestine. Moreover, *B. coagulans* and *L. plantarum* lack the filamentous growth habit of *A. niger*, which physically penetrates and disrupts the rigid cell wall matrix of cottonseed meal, thereby exposing encapsulated proteins to digestive enzymes [57]. Inadequate fermentation with these bacterial strains would thus fail to effectively degrade both the protein matrix and the cell wall–polysaccharide complexes, limiting access of digestive enzymes to peptide bonds and reducing amino acid release at the terminal ileum [58]. This mechanistic interpretation is further supported by the significantly lower AID of Cys, a sulfur-containing amino acid often embedded within the protein matrix and thus less accessible to enzymatic hydrolysis [59]. In summary, the present results demonstrate that fermentation efficacy varies substantially depending on the microbial strain and process conditions employed. The superior performance of *A. niger* fermentation (Group 3) underscores the importance of selecting strains with potent proteolytic activity and the ability to disrupt the structural integrity of the feed matrix. Future research should investigate the specific biochemical transformations induced by different fermentation regimes, with particular emphasis on alterations in peptide profiles, the degradation patterns of anti-nutritional factors, and changes in protein solubility and structure.

### 4.4. Predictive Equations for Available Energy and SID AAs of FCSM and FCSP for Growing Pigs

The DE of FCSM and FCSP ranged broadly from 12.58 to 21.42 MJ/kg DM. Among all fermentation batches, F2 exhibited the lowest DE value of 12.58 MJ/kg DM, which coincided with its markedly suppressed apparent total tract digestibility (ATTD) of dry matter (DM), organic matter (OM) and gross energy (GE). This phenomenon can be attributed to insufficient lignocellulose degradation under the fermentation regime of F2. Recalcitrant cellulose and lignin-rich structural carbohydrates remained largely intact after fermentation. Such indigestible fibrous fractions cannot be hydrolyzed by porcine endogenous digestive enzymes in the small intestine, and their rigid three-dimensional matrix physically entraps intracellular energy substrates [60]. Consequently, substantial amounts of unutilized energy bound to fiber were excreted via feces, resulting in pronounced elevation of fecal energy loss and the subsequent reduction in overall DE [61]. Conversely, F6 and F9 attained the greatest DE levels (20.79 and 21.42 MJ/kg DM, respectively), concurrent with superior ATTD of organic nutrients. Sufficient hydrolytic breakdown of fibrous polysaccharides and bioconversion of native cottonseed protein into readily absorbable small peptides synergistically improved the energy utilization efficiency of these two fermented batches. The ME value ranged from 11.90 to 19.31 MJ/kg DM. The ME/DE conversion ratio fell within a narrow window of 94.06 to 96.64% for all fermented substrates. Such consistent post-absorptive energy partitioning reveals that microbial fermentation predominantly modulates fecal energy loss, a parameter quantified by DE, rather than altering urinary nitrogen excretion or intestinal gaseous energy dissipation after nutrient absorption at 11.90 and 19.31 MJ/kg DM. The ME/DE conversion ratio fell within a narrow window of 94.06 to 96.64% for all fermented substrates. Such consistent post-absorptive energy partitioning reveals that microbial fermentation predominantly modulates fecal energy loss, a parameter quantified by DE, rather than altering urinary nitrogen excretion or intestinal gaseous energy dissipation after nutrient absorption.

In the present study, CP exhibited an extremely significant positive correlation with both DE and ME. In contrast, NDF and ADF exerted strong adverse effects on DE and ME, as lignocellulosic fractions are inherently indigestible in the porcine small intestine and their intricate three-dimensional architecture physically entraps intracellular nutrients, impeding the accessibility of digestive enzymes to energy substrates [62]. For SID Lys, contrasting correlation patterns were observed. It was negatively correlated with CP and Ash, but positively correlated with OM, NDF, and ADF. The negative CP-Lys association is mechanistically attributed to the Maillard-type condensation between the ε-amino group of lysine and the aldehyde moieties of free gossypol, forming high-molecular-weight indigestible Schiff-base adducts that resist intestinal proteolysis [41]. Conversely, the positive correlation with fiber fractions reflects a unique feature of fermented feedstuffs. Controlled fiber hydrolysis during fermentation disrupts the compact protein–fiber matrix, thereby improving the steric accessibility of lysine residues to pancreatic proteases and enhancing ileal digestibility [40]. SID Trp and SID Met exhibited highly consistent correlation patterns. Both were positively correlated with CP and Ash and negatively correlated with NDF, ADF, and free gossypol. Elevated CP provides an expanded pool of proteinaceous precursors for these amino acids; mineral elements in ash may serve as essential cofactors for microbial proteases during fermentation, facilitating the release of sulfur-containing and aromatic amino acid-rich peptides [38]. Lignocellulosic NDF and ADF act as physical barriers enclosing intracellular proteins, whereas free gossypol exhibits high binding affinity for sulfur-containing (methionine) and aromatic (tryptophan) amino acids through non-covalent interactions and Schiff-base formation [62], forming stable indigestible complexes that markedly reduce their ileal bioavailability. These trends were mirrored in the regression coefficients, with CP positive and fiber/gossypol negative. SID Met and SID Trp were also positively correlated with each other, supporting their parallel modeling. Notably, no routine component showed significant correlation with SID Thr, indicating that threonine digestibility is governed by multiple complex factors—including fermentation strain specificity, protein secondary structure conformation, and peptide chain length distribution—rather than any single conventional chemical index. This intrinsic complexity also explains the comparatively lower coefficient of determination of the Thr prediction model relative to the other amino acid equations. Collectively, all established DE, ME, and SID amino acid prediction models showed acceptable fit and statistical significance. These equations enable rapid estimation of available energy and digestible amino acids from conventional chemical data, circumventing the need for costly and labor-intensive in vivo trials, and thereby providing robust technical support for precision diet formulation in growing pigs fed fermented cottonseed products. However, due to the limited sample size (n = 10) and lack of external validation, they should be viewed as preliminary. Independent validation with a broader range of fermented cottonseed products is needed before practical application.

## 5. Conclusions

In the present study, 10 representative fermented cottonseed substrates were selected from 103 fermented batches using multivariate statistical methods. Short-term aerobic fermentation inoculated with Aspergillus niger (Treatment F3) produced the best comprehensive performance under the conditions tested in this study, including effective fiber breakdown, efficient free gossypol detoxification, and the highest nutrient and amino acid digestibility in growing pigs. DE was a sensitive indicator to distinguish fermentation quality, while ME remained stable across all treatments. CP, NDF and ADF were strong predictors of DE and ME. Distinct correlation trends existed for different SID AAs, and no single conventional index could well predict SID Thr. The established multiple linear regression models (R^2^ = 0.57–0.93) allow rapid estimation of available energy and digestible amino acids through routine chemical testing, which may reduce the reliance on costly in vivo trials. However, due to the limited sample size (n = 10) and lack of external validation, these equations should be viewed as preliminary. Independent validation with a broader range of fermented cottonseed products is needed before they can be recommended for practical feed formulation.

## Figures and Tables

**Table 1:** Nutrient components of CSM and CSP samples (%, on a DM basis).

Items	C1	C2	C3	C4	C5
Ash	7.71	8.42	8.12	7.67	6.78
OM	91.78	91.63	91.65	92.33	93.22
CP	61.68	59.91	53.16	48.11	46.51
NDF	21.28	10.5	23.77	17.08	25.66
ADF	7.48	6.53	17.07	9.02	16.53
GE (MJ/kg)	19.88	19.83	19.61	20.3	19.74
FG (mg/kg)	296.50	594.12	692.07	1493.73	773.33

Note: Each value represents the mean of duplicate determinations. Ash, crude ash; OM, organic matter; CP, crude protein; NDF, neutral detergent fiber; ADF, acid detergent fiber; GE, gross energy; FG, Free gossypol.

**Table 2:** The parameters for aerobic fermentation.

Aerobic Fermentation				
Cottonseed Meal (g)	Water Supplementation (mL)	Culture (g)	Temperature (°C)	Time (h)
100	60	A (0.2)	30	24
100	60	B (0.2)	30	24
100	60	C (0.2)	30	48
100	60	D (0.05)	37	24

Note: *Aspergillus oryzae* (A); *Aspergillus niger* (B); *Saccharomyces cerevisiae* (C); *Bacillus subtilis* (D).

**Table 3:** The parameters for anaerobic fermentation.

Anaerobic Fermentation				
Cottonseed Meal (g)	Water Supplementation (mL)	Culture (g)	Temperature (°C)	Time (h)
100	60	D (0.05) + C (0.2)	37	72
100	60	E (0.6) + C (0.2)	37	72
100	60	F (0.1) + C (0.2)	37	72
100	60	D (0.05) + E (0.6)	37	72
100	60	D (0.05) + F (0.1)	37	72
100	60	E (0.6) + F (0.1)	37	72
100	60	C (0.2) + D (0.05) + E (0.6)	37	72
100	60	C (0.2) + D (0.05) + F (5 mL)	37	72
100	60	C (0.2) + E (0.6) + F (5 mL)	37	72
100	60	D (0.2) + E (0.6) + F (5 mL)	37	72
100	60	C (0.2) + D (0.05) + E (0.6) + F (5 mL)	37	72
100	60	D (0.05) + C (0.2) + G (0.1)	37	72
100	60	E (0.6) + C (0.2) + G (0.1)	37	72
100	60	F (5 mL) + C (0.2) + G (0.1)	37	72
100	60	D (0.05) + E (0.6) + G (0.1)	37	72
100	60	D (0.05) + F (5 mL) + G (0.1)	37	72
100	60	E (0.6) + F (5 mL) + G (0.1)	37	72
100	60	E (0.6) + F (5 mL) + C (0.2) + D (0.05) + G (0.1)	37	72

Note: *Saccharomyces cerevisiae* (C); *Bacillus subtilis* (D); *Bacillus coagulans* (E); *Lactobacillus plantarum* (F); *Neutral protease* (G).

**Table 4:** Nutritional values of FCSM and FCSP samples (%, on a DM basis).

Items	Min	Max	Mean	SD	CV
Ash	6.52	9.84	7.98	0.89	11.15
OM	90.16	93.48	92.02	0.89	0.97
NDF	7.39	50.97	27.7	10.61	38.30
ADF	3.33	25.72	14.12	6.64	47.01
CP	46.75	76.41	59.71	8.74	14.63
GE (MJ/kg)	14.92	21.91	19.69	0.72	3.67
FG (mg/kg)	212.39	1152.39	549.85	249.10	45.30
Small peptide	4.88	31.22	14.82	7.05	47.59
Protein solubility	22.93	95.28	64.16	21.74	33.88

Note: Ash, crude ash; OM, organic matter; CP, crude protein; NDF, neutral detergent fiber; ADF, acid detergent fiber; GE, gross energy; FG, free gossypol.

**Table 5:** The fermentation parameters of 10 representative FCSM and FCSP samples.

**Aerobic fermentation**						
**FCSM/FCSP**	**CSM/CSP**	**Substrate Amount (kg)**	**Culture (kg)**	**Water (L)**	**Temperature (°C)**	**Time (h)**
F1	C1	50	C (0.1)	30	30	48
F2	C3	50	C (0.1)	30	30	48
F3	C2	50	B (0.1)	30	30	24
F4	C3	50	B (0.1)	30	30	24
F5	C1	50	D (0.025)	30	37	24
**Anaerobic fermentation**						
F6	C1	50	D (0.025) + F (0.05) + G (0.05)	30	37	72
F7	C5	50	E (0.3) + F (0.05)	30	37	72
F8	C3	50	D (0.025) + C (0.1)	30	37	72
F9	C2	50	D (0.025) + C (0.1) + G (0.05)	30	37	72
F10	C5	50	E (0.3) + F (0.05) + C (0.1) + D (0.025) + G (0.05)	30	37	72

Note: *Aspergillus niger* (B); *Saccharomyces cerevisiae* (C); *Bacillus subtilis* (D); *Bacillus coagulans* (E); *Lactobacillus plantarum* (F); *Neutral protease* (G).

**Table 6:** Nutrient compositions of 10 representative FCSM and FCSP samples (%, on a DM basis).

Items	Representative Samples										CV
	F1	F2	F3	F4	F5	F6	F7	F8	F9	F10	
Ash	8.92	6.99	8.99	7.31	8.16	8.67	7.03	7.04	8.43	7.33	10.49
OM	91.08	93.01	91.01	92.69	91.84	91.33	92.97	92.96	91.57	92.67	0.76
CP	62.83	40.58	63.66	42.06	59.57	62.76	42.87	42.72	59.65	44.45	19.89
NDF	10.61	35.79	9.9	33.34	10.26	9.09	33.84	35.17	9.66	34.87	59.58
ADF	4.91	21.4	5.31	21.25	5.72	4.91	20.89	20.18	5.53	20.36	62.80
GE (MJ/kg)	20.15	19.75	20.07	19.76	19.88	19.96	19.34	20.6	19.64	19.34	1.91
FG (mg/kg)	264.82	668.31	213.14	489.28	238.29	222.53	531.76	472.75	450.19	290.86	41.19

Note: Ash, crude ash; OM, organic matter; CP, crude protein; NDF, neutral detergent fiber; ADF, acid detergent fiber; GE, gross energy; FG, free gossypol.

**Table 7:** Composition and nutritional contents of all diets for Exp. 2 (%).

Items	Basal Diet	Diets									
		1	2	3	4	5	6	7	8	9	10
Corn	96.9	71.9	71.9	71.9	71.9	71.9	71.9	71.9	71.9	71.9	71.9
FCSM/FCSP	0.0	25.0	25.0	25.0	25.0	25.0	25.0	25.0	25.0	25.0	25.0
Limestone	0.6	0.6	0.6	0.6	0.6	0.6	0.6	0.6	0.6	0.6	0.6
Dicalcium phosphate	1.7	1.7	1.7	1.7	1.7	1.7	1.7	1.7	1.7	1.7	1.7
Salt	0.3	0.3	0.3	0.3	0.3	0.3	0.3	0.3	0.3	0.3	0.3
Premix ^1^	0.5	0.5	0.5	0.5	0.5	0.5	0.5	0.5	0.5	0.5	0.5
Total ^2^	100.0	100.0	100.0	100.0	100.0	100.0	100.0	100.0	100.0	100.0	100.0
Analysis nutrient levels ^3^											
Moisture	9.86	10.73	8.76	9.46	9.81	8.95	13.45	9.15	10.82	13.55	8.30
Ash	4.05	6.05	7.42	6.05	6.09	5.75	5.74	5.47	5.33	6.20	5.75
OM	95.95	93.95	92.58	93.95	93.91	94.25	94.26	94.53	94.67	93.80	94.25
CP	8.09	22.40	19.22	22.63	19.11	22.19	19.67	18.05	17.22	20.39	19.07
NDF	11.66	7.58	17.50	11.69	16.15	11.90	12.22	17.38	17.36	12.02	17.74
ADF	3.67	4.13	7.22	3.51	6.11	3.54	3.67	7.35	7.20	3.82	7.17
GE (MJ/kg)	18.04	18.43	18.15	18.52	18.48	18.57	18.54	18.54	18.39	18.32	18.37

Note: ^1^ Premix provided per kilogram of complete feed: vitamin A 5512 IU, vitamin E 30 IU, vitamin D3 2200 IU, vitamin K3 2.2 mg, riboflavin 4.0 mg, niacin 30.0 mg, pantothenic acid 14.0 mg, folic acid 0.7 mg, choline chloride 400.0 mg, thiamine 1.5 mg, pyridoxine 3.0 mg, vitamin B12, 27.6 μg, biotin 44.0 μg, Fe 75.0 mg, Cu 100.0 mg, Zn 75.0 mg, Mn 40.0 mg, Se 0.3 mg, I 0.3 mg. ^2^ Composition content of all diets was on a fed basis. ^3^ Analysis nutrient levels were on a DM basis. Ash, crude ash; OM, organic matter; CP, crude protein; NDF, neutral detergent fiber; ADF, acid detergent fiber; GE, gross energy.

**Table 8:** Composition and nutritional content of the diets in Exp. 3 (%, as fed basis).

Items	Nitrogen-Free Diet	Test Diet
Corn starch	79.2	54.5
FCSM/FCSP	0.0	25.0
Soybean oil	3.3	3.3
Sucrose	10.0	10.0
Cellulose acetate	4.0	4.0
Dicalcium phosphate	1.6	1.6
Limestone	0.5	0.5
Salt	0.3	0.3
Chromic oxide	0.3	0.3
Potassium carbonate	0.3	0.0
Premix ^1^	0.5	0.5
Total	100.0	100.0

Note: ^1^ Premix provided per kilogram of complete feed: vitamin A 5512 IU, vitamin E 30 IU, vitamin D3 2200 IU, vitamin K3 2.2 mg, riboflavin 4.0 mg, niacin 30.0 mg, pantothenic acid 14.0 mg, folic acid 0.7 mg, choline chloride 400.0 mg, thiamine 1.5 mg, pyridoxine 3.0 mg, vitamin B12, 27.6 μg, biotin 44.0 μg, Fe 75.0 mg, Cu 100.0 mg, Zn 75.0 mg, Mn 40.0 mg, Se 0.3 mg, I 0.3 mg.

**Table 9:** Nutritional content of all diets for Exp. 3 (%, as fed basis).

Items	Nitrogen-Free Diet	Test Diets									
		1	2	3	4	5	6	7	8	9	10
DM	90.60	89.75	91.52	90.40	90.25	91.28	88.15	91.54	89.76	87.78	91.78
CP	3.60	13.26	12.70	17.46	12.69	16.17	15.02	12.78	11.56	12.14	11.59
Essential AA											
Arg	0.01	0.71	1.83	2.35	0.88	1.13	1.65	0.92	0.94	1.09	1.14
His	0.00	0.18	0.51	0.65	0.18	0.27	0.40	0.25	0.26	0.24	0.30
Ile	0.00	0.22	0.62	0.81	0.29	0.32	0.47	0.30	0.30	0.35	0.35
Leu	0.02	0.39	1.16	1.50	0.52	0.62	0.84	0.54	0.54	0.62	0.64
Lys	0.00	0.25	0.77	1.00	0.37	0.40	0.56	0.34	0.35	0.39	0.46
Met	0.71	0.16	0.12	0.16	0.12	0.22	0.17	0.15	0.09	0.12	0.15
Phe	0.00	0.37	1.04	1.36	0.48	0.56	0.8	0.50	0.50	0.59	0.59
Thr	0.00	0.20	0.57	0.74	0.28	0.31	0.42	0.26	0.28	0.31	0.32
Trp	0.14	0.14	0.11	0.11	0.10	0.18	0.14	0.13	0.11	0.14	0.12
Val	0.00	0.32	0.91	1.16	0.43	0.48	0.67	0.42	0.44	0.49	0.51
Non-essential AA											
Ala	0.02	0.30	0.85	0.97	0.39	0.42	0.59	0.35	0.38	0.44	0.45
Asp	0.03	0.61	1.76	2.31	0.81	0.97	1.32	0.81	0.85	0.97	1.02
Cys	0.01	0.21	0.19	0.28	0.17	0.27	0.18	0.17	0.13	0.20	0.14
Glu	0.26	1.36	3.92	5.11	1.67	2.07	2.81	1.74	1.79	2.06	2.16
Gly	0.00	0.25	0.72	0.90	0.33	0.36	0.6	0.31	0.34	0.38	0.46
Pro	0.05	0.30	0.87	1.09	0.41	0.50	0.36	0.43	0.43	0.51	0.29
Ser	0.01	0.26	0.73	0.95	0.36	0.40	0.49	0.34	0.35	0.40	0.38
Tyr	0.00	0.13	0.17	0.29	0.18	0.14	0.34	0.21	0.21	0.25	0.25

Note: DM, dry matter; CP, crude protein; AA, amino acid; Arg, arginine; His, histidine; Leu, leucine; Ile, isoleucine; Lys, lysine; Met, methionine; Phe, phenylalanine; Thr, threonine; Trp, tryptophan; Val, valine; Ala, alanine; Asp, aspartic; Cys, cystine; Gly, glycine; Pro, proline; Ser, serine; Tyr, tyrosine.

**Table 10:** Conventional chemical compositions of ingredient (C1) and fermented samples (F1, F5, F6).

Items	C1	F1	F5	F6	SEM	*p* Value
OM	91.78	91.54	91.47	91.88	0.14	0.69
DM	93.32 ^a^	86.88 ^b^	92.72 ^a^	80.84 ^b^	0.10	<0.01
CP	61.68 ^b^	62.82 ^a^	59.64 ^c^	63.01 ^a^	0.09	<0.01
NDF	21.28 ^a^	10.38 ^b^	10.25 ^b^	9.08 ^b^	0.18	<0.01
ADF	7.48	4.89	4.86	4.99	0.27	0.03
GE (MJ/kg)	19.88	20.11	19.91	19.90	0.03	0.05
FG (mg/kg)	296.50 ^a^	264.82 ^b^	238.29 ^c^	222.53 ^d^	0.42	<0.01

Note: OM, organic matter; DM, dry matter; CP, crude protein; NDF, neutral detergent fiber; ADF, acid detergent fiber; GE, gross energy; FG, Free gossypol. Means within the same row with different lowercase letters are significantly different (*p* < 0.05). SEM = standard error of the mean.

**Table 11:** Conventional chemical compositions of ingredient (C2) and fermented samples (F3, F9).

Items	C2	F3	F9	SEM	*p* Value
OM	91.63 ^a^	90.97 ^a^	79.89 ^b^	0.11	<0.01
DM	92.63 ^a^	89.50 ^b^	77.70 ^c^	0.36	<0.01
CP	59.91 ^b^	63.57 ^a^	51.48 ^c^	0.05	<0.01
NDF	10.50	9.94	9.57	0.42	0.674
ADF	6.53	5.33	4.47	0.11	<0.01
GE (MJ/kg)	19.83	20.05	17.86	0.30	0.049
FG (mg/kg)	594.12 ^a^	213.14 ^b^	450.19 ^a^	23.09	<0.01

Note: OM, organic matter; DM, dry matter; CP, crude protein; NDF, neutral detergent fiber; ADF, acid detergent fiber; GE, gross energy; FG, free gossypol. Means within the same row with different lowercase letters are significantly different (*p* < 0.05). SEM = standard error of the mean.

**Table 12:** Conventional chemical compositions of ingredient (C3) and fermented samples (F2, F4, F8).

Items	C3	F2	F4	F8	SEM	*p* Value
OM	91.65	92.51	91.79	92.51	0.15	0.16
DM	91.14 ^ab^	93.04 ^a^	89.37 ^b^	86.65 ^c^	0.22	<0.01
CP	53.16	46.30	46.44	47.48	2.20	0.66
NDF	23.77	31.74	29.57	32.32	1.43	0.22
ADF	17.07	19.98	18.97	19.01	0.71	0.56
GE (MJ/kg)	19.61	19.86	20.20	19.98	0.15	0.60
FG (mg/kg)	692.07 ^a^	668.31 ^b^	489.28 ^c^	472.75 ^d^	0.26	<0.01

Note: OM, organic matter; DM, dry matter; CP, crude protein; NDF, neutral detergent fiber; ADF, acid detergent fiber; GE, gross energy; FG, free gossypol. Means within the same row with different lowercase letters are significantly different (*p* < 0.05). SEM = standard error of the mean.

**Table 13:** Conventional chemical compositions of ingredient (C5) and fermented samples (F7, F10).

Items	C5	F7	F10	SEM	*p* Value
OM	93.22 ^a^	93.00 ^b^	92.71 ^c^	0.01	<0.01
DM	91.72	91.94	91.64	0.44	1.0
CP	46.51	45.64	46.83	1.24	0.92
NDF	25.66 ^b^	34.10 ^a^	31.41 ^ab^	0.93	0.04
ADF	16.53 ^b^	20.00 ^a^	19.12 ^ab^	0.39	0.04
GE (MJ/kg)	19.74 ^a^	19.43 ^b^	19.41 ^b^	0.04	0.02
FG (mg/kg)	773.33 ^a^	531.76 ^b^	290.86 ^c^	19.16	<0.01

Note: OM, organic matter; DM, dry matter; CP, crude protein; NDF, neutral detergent fiber; ADF, acid detergent fiber; GE, gross energy; FG, free gossypol. Means within the same row with different lowercase letters are significantly different (*p* < 0.05). SEM = standard error of the mean.

**Table 14:** The ATTD of nutrients and available energy in the basal diet and test diets of growing pigs in Exp. 2 (%).

Items	Basal Diet	Test Diets										SEM	*p* Value
		1	2	3	4	5	6	7	8	9	10		
DM	91.20 ^a^	88.96 ^a^	81.18 ^b^	87.21 ^ab^	85.67 ^ab^	88.98 ^a^	90.87 ^a^	84.74 ^ab^	85.15 ^ab^	90.97 ^a^	84.37 ^ab^	0.48	<0.001
OM	92.65 ^a^	91.00 ^ab^	83.79 ^c^	89.46 ^abc^	87.61 ^abc^	90.91 ^ab^	92.66 ^a^	86.50 ^abc^	87.00 ^abc^	92.65 ^a^	86.12 ^bc^	0.40	<0.001
CP	81.34 ^ab^	87.24 ^ab^	80.37 ^b^	89.20 ^ab^	85.66 ^ab^	88.29 ^ab^	90.48 ^a^	84.48 ^ab^	84.21 ^ab^	90.68 ^a^	83.11 ^ab^	0.61	0.004
NDF	65.86	55.60	51.45	64.13	60.15	72.03	72.44	58.03	56.24	72.96	57.49	1.61	0.051
ADF	62.59 ^ab^	48.41 ^ab^	40.23 ^b^	49.98 ^ab^	39.71 ^b^	60.80 ^ab^	62.86 ^ab^	48.78 ^ab^	43.95 ^ab^	70.51 ^a^	50.66 ^ab^	1.80	0.007
GE	90.84 ^a^	89.45 ^a^	82.04 ^b^	88.07 ^ab^	85.98 ^ab^	89.35 ^a^	91.59 ^a^	84.95 ^ab^	85.42 ^ab^	91.33 ^a^	84.50 ^ab^	0.46	<0.001
DE (MJ/kg DM)	16.18 ^ab^	16.21 ^a^	14.89 ^b^	16.31 ^a^	15.89 ^ab^	16.59 ^a^	16.99 ^a^	15.70 ^ab^	15.71 ^ab^	17.00 ^a^	15.64 ^ab^	0.09	<0.01
ME (MJ/kg DM)	15.69 ^ab^	15.66 ^ab^	14.45 ^b^	15.28 ^ab^	15.41 ^ab^	16.01 ^ab^	16.33 ^a^	15.19 ^ab^	14.61 ^ab^	16.18 ^ab^	15.15 ^ab^	0.11	0.016
ME/DE	96.96	96.60	96.99	93.56	96.96	96.52	96.1	96.72	92.84	95.09	96.89	0.36	0.221

Note: DM, dry matter; OM, organic matter; CP, crude protein; NDF, neutral detergent fiber; ADF, acid detergent fiber; GE, gross energy; DE, digestible energy; ME, metabolizable energy. Means within the same row with different lowercase letters are significantly different (*p* < 0.05). SEM = standard error of the mean.

**Table 15:** The ATTD of nutrients and available energy in the FCSM and FCSP ingredients for growing pigs, calculated by the substitution method in Exp. 2 (%).

Items	Test Diets										SEM	*p* Value
	1	2	3	4	5	6	7	8	9	10		
DM	68.74 ^ab^	51.14 ^b^	75.25 ^a^	69.09 ^ab^	82.34 ^a^	78.23 ^a^	65.36 ^ab^	67.04 ^ab^	77.01 ^a^	63.91 ^ab^	1.56	0.02
OM	75.03 ^ab^	57.21 ^b^	79.90 ^a^	72.49 ^ab^	85.67 ^a^	83.31 ^a^	68.06 ^ab^	70.03 ^ab^	82.09 ^a^	66.51 ^ab^	1.33	<0.01
CP	76.73 ^ab^	64.72 ^b^	96.58 ^a^	82.83 ^ab^	90.77 ^ab^	93.38 ^a^	81.19 ^ab^	70.26 ^ab^	87.16 ^ab^	75.71 ^ab^	1.73	0.03
GE	68.63 ^ab^	51.91 ^b^	76.02 ^a^	67.63 ^ab^	81.11 ^a^	79.56 ^a^	63.54 ^ab^	65.43 ^ab^	76.39 ^a^	61.73 ^ab^	1.51	0.01
DE (MJ/kg DM)	18.34 ^ab^	12.58 ^b^	19.18 ^a^	17.38 ^ab^	20.25 ^a^	20.79 ^a^	16.15 ^ab^	17.35 ^ab^	21.42 ^a^	16.83 ^ab^	0.42	<0.01
ME (MJ/kg DM)	17.34	11.9	16.13	16.65	18.78	19.31	15.33	16.20	18.65	15.88	0.51	0.09
ME/DE	95.95	94.35	94.81	95.82	94.54	94.06	95.81	94.79	94.24	96.64	0.92	0.24

Note: DM, dry matter; OM, organic matter; CP, crude protein; GE, gross energy; DE, digestible energy; ME, metabolizable energy. Means within the same row with different lowercase letters are significantly different (*p* < 0.05). SEM = standard error of the mean.

**Table 16:** The AID of AAs of FCSM and FCSP of growing pigs in Exp. 3 (%).

Items	Test Diets										SEM	*p* Value
	1	2	3	4	5	6	7	8	9	10		
Essential AA												
Met	82.20 ^ab^	75.81 ^ab^	83.68 ^a^	72.91 ^ab^	82.25 ^ab^	80.13 ^ab^	76.17 ^ab^	68.36 ^b^	78.28 ^ab^	81.81 ^ab^	0.98	0.03
Trp	68.23	69.71	64.61	54.34	71.72	69.73	60.66	68.66	71.46	65.89	1.26	0.11
Leu	51.85 ^e^	84.97 ^ab^	87.96 ^a^	60.19 ^de^	63.51 ^cde^	75.58 ^abc^	66.80 ^cde^	75.21 ^abcd^	69.24 ^cd^	71.86 ^bcd^	1.01	<0.01
Ile	51.52 ^d^	84.91 ^a^	87.10 ^a^	63.79 ^bcd^	59.18 ^cd^	74.46 ^ab^	65.87 ^bcd^	74.12 ^ab^	69.50 ^bc^	69.74 ^bc^	0.98	<0.01
Arg	82.41 ^b^	93.90 ^ab^	95.09 ^a^	85.11 ^ab^	88.02 ^ab^	91.63 ^ab^	83.47 ^ab^	90.41 ^ab^	90.06 ^ab^	88.92 ^ab^	0.35	<0.01
His	70.24 ^cd^	88.57 ^ab^	91.96 ^a^	67.70 ^d^	77.57 ^bcd^	83.80 ^ab^	71.34 ^cd^	84.52 ^ab^	78.28 ^bcd^	81.27 ^abc^	0.82	<0.01
Val	54.33 ^c^	85.94 ^a^	88.48 ^a^	63.49 ^bc^	64.67 ^bc^	76.39 ^ab^	67.07 ^bc^	76.55 ^ab^	69.22 ^bc^	72.96 ^ab^	1.05	<0.01
Thr	32.91 ^e^	75.44 ^ab^	83.42 ^a^	50.55 ^cd^	47.26 ^de^	65.20 ^bc^	50.43 ^cd^	63.92 ^bcd^	57.51 ^cd^	61.23 ^bcd^	1.12	<0.01
Phe	69.63 ^d^	90.17 ^ab^	92.56 ^a^	75.46 ^cd^	76.45 ^cd^	84.51 ^abc^	73.07 ^cd^	84.14 ^abc^	80.33 ^bcd^	81.90 ^abc^	0.80	<0.01
Lys	27.32 ^b^	80.94 ^ab^	81.87 ^a^	54.15 ^ab^	40.60 ^ab^	65.21 ^ab^	45.17 ^ab^	66.57 ^ab^	57.07 ^ab^	64.27 ^ab^	1.10	<0.01
Non-essential AA												
Cys	80.80 ^ab^	80.72 ^ab^	84.63 ^a^	75.83 ^ab^	81.64 ^ab^	74.29 ^ab^	64.04 ^b^	72.97 ^ab^	76.75 ^ab^	68.62 ^ab^	1.29	0.03
Ala	29.76 ^e^	78.18 ^a^	80.33 ^a^	54.27 ^bcd^	45.59 ^de^	66.52 ^abc^	49.08 ^bc^	68.34 ^ab^	58.88 ^bcd^	63.05 ^abcd^	1.16	<0.01
Ser	50.80 ^d^	83.59 ^ab^	88.04 ^a^	63.79 ^c^	63.30 ^c^	71.80 ^bc^	62.24 ^cd^	72.72 ^bc^	70.04 ^c^	68.78 ^c^	0.81	<0.01
Asp	58.82 ^d^	86.24 ^ab^	90.19 ^a^	68.63 ^cd^	70.64 ^c^	78.34 ^bc^	70.33 ^c^	78.48 ^bc^	75.11 ^c^	75.96 ^bc^	0.70	<0.01
Gly	25.32 ^d^	70.63 ^ab^	77.73 ^a^	41.22 ^cd^	44.17 ^bcd^	64.07 ^abc^	38.15 ^cd^	59.47 ^abc^	48.93 ^bcd^	58.11 ^abc^	1.77	<0.01
Pro	61.91	67.76	64.01	54.89	44.02	60.68	63.49	81.59	80.50	70.27	2.53	0.06
Glu	73.49 ^c^	91.93 ^a^	93.45 ^a^	78.43 ^bc^	80.29 ^bc^	86.22 ^ab^	77.39 ^bc^	86.88 ^ab^	83.95 ^ab^	84.26 ^ab^	0.67	<0.01
Tyr	52.00 ^cd^	57.73 ^bcd^	81.45 ^a^	64.74 ^abcd^	47.86 ^d^	81.31 ^a^	70.00 ^abc^	80.81 ^a^	77.68 ^ab^	76.79 ^ab^	1.36	<0.01

Note: AA, amino acid; Arg, arginine; His, histidine; Leu, leucine; Ile, Isoleucine; Lys, Lysine; Met, methionine; Phe, phenylalanine; Thr, threonine; Trp, tryptophan; Val, valine; Ala, alanine; Asp, aspartic; Cys, cystine; Gly, glycine; Pro, proline; Ser, serine; Tyr, tyrosine. Means within the same row with different lowercase letters are significantly different (*p* < 0.05). SEM = standard error of the mean.

**Table 17:** The SID of AAs of FCSM and FCSP of growing pigs in Exp. 3 (%).

Items	Test Diets										SEM	*p* Value
	1	2	3	4	5	6	7	8	9	10		
Essential AA												
Met	85.92	81.07	87.54	78.15	85.00	83.52	80.35	75.17	83.04	86.00	0.98	0.17
Trp	75.29	74.70	73.74	64.78	77.27	76.55	68.63	77.48	78.54	74.66	1.20	0.31
Leu	64.59 ^d^	89.34 ^ab^	91.29 ^a^	69.92 ^cd^	71.64 ^cd^	81.39 ^abc^	76.16 ^bcd^	84.40 ^abc^	77.04 ^abcd^	79.84 ^abc^	<0.01	<0.01
Ile	63.68 ^c^	86.66 ^a^	90.42 ^a^	67.96 ^bc^	67.65 ^bc^	80.09 ^ab^	74.84 ^abc^	83.03 ^ab^	77.05 ^abc^	77.59 ^abc^	1.06	<0.01
Arg	87.59 ^d^	95.95 ^ab^	96.67 ^a^	89.30 ^cd^	91.33 ^bcd^	93.81 ^abc^	87.56 ^d^	94.34 ^abc^	93.36 ^abc^	92.21 ^abcd^	0.35	<0.01
His	75.58 ^cd^	90.54 ^ab^	93.49 ^a^	73.22 ^d^	81.22 ^bcd^	86.23 ^ab^	81.34 ^bcd^	88.30 ^ab^	82.21 ^bcd^	84.58 ^abc^	0.63	<0.01
Val	66.98 ^c^	87.86 ^ab^	92.02 ^a^	72.96 ^bc^	73.28 ^bc^	82.37 ^abc^	76.99 ^abc^	85.94 ^ab^	77.34 ^abc^	81.20 ^abc^	1.03	<0.01
Thr	53.83 ^c^	82.96 ^ab^	89.12 ^a^	65.43 ^bc^	61.23 ^bc^	75.05 ^bc^	66.73 ^bc^	78.88 ^ab^	70.82 ^bc^	74.47 ^bc^	1.12	<0.01
Phe	77.82 ^d^	93.11 ^ab^	94.77 ^a^	81.78 ^cd^	81.86 ^cd^	88.18 ^abc^	84.93 ^bcd^	90.09 ^abc^	85.30 ^bcd^	87.11 ^abc^	0.61	<0.01
Lys	38.63 ^e^	84.73 ^a^	84.76 ^a^	61.94 ^bcd^	47.94 ^de^	70.22 ^bc^	59.96 ^cd^	74.77 ^ab^	64.19 ^bc^	70.67 ^abc^	0.96	<0.01
Non-essential AA												
Cys	87.16	87.72	89.44	83.53	86.55	81.34	72.17	83.08	83.20	78.09	1.29	0.17
Ala	47.53 ^d^	84.56 ^ab^	85.87 ^a^	68.02 ^bc^	58.51 ^cd^	75.46 ^abc^	64.45 ^cd^	82.23 ^ab^	70.63 ^abc^	75.26 ^abc^	1.16	<0.01
Ser	64.27 ^d^	88.50 ^ab^	91.77 ^a^	73.61 ^cd^	72.24 ^cd^	78.92 ^bc^	72.94 ^cd^	82.74 ^abc^	78.68 ^bc^	78.14 ^bc^	0.81	<0.01
Asp	69.87 ^d^	90.12 ^ab^	93.11 ^a^	76.96 ^cd^	77.69 ^cd^	83.32 ^abc^	78.70 ^cd^	86.32 ^abc^	81.84 ^bc^	82.64 ^abc^	0.70	<0.01
Gly	51.63 ^b^	80.03 ^a^	87.90 ^a^	69.23 ^ab^	69.89 ^ab^	78.94 ^a^	68.42 ^ab^	85.79 ^a^	72.54 ^ab^	78.44 ^a^	1.74	<0.01
Pro	66.89	80.16	79.30	68.62	78.14	65.62	74.94	94.39	86.29	65.36	3.40	0.57
Glu	79.27 ^d^	93.97 ^ab^	95.00 ^a^	83.16 ^cd^	84.15 ^cd^	88.96 ^abc^	86.22 ^bcd^	91.27 ^abc^	87.68 ^abcd^	87.98 ^abc^	0.56	<0.01
Tyr	66.91 ^bc^	76.94 ^abc^	88.03 ^a^	75.19 ^abc^	62.12 ^c^	86.75 ^a^	79.33 ^ab^	89.66 ^a^	85.23 ^a^	84.56 ^a^	1.05	<0.01

Note: AA, amino acid; Arg, arginine; His, histidine; Leu, leucine; Ile, isoleucine; Lys, lysine; Met, methionine; Phe, phenylalanine; Thr, threonine; Trp, tryptophan; Val, valine; Ala, alanine; Asp, aspartic; Cys, cystine; Gly, glycine; Pro, proline; Ser, serine; Tyr, tyrosine. Means within the same row with different lowercase letters are significantly different (*p* < 0.05). SEM = standard error of the mean.

**Table 18:** Correlation analysis of routine chemical compositions and available energy values of ten FCSM and FCSP samples fed to growing pigs.

Items	DE	ME	Ash	OM	CP	NDF	ADF	GE	DM
DE	1								
ME	0.96 **	1							
Ash	0.74 *	0.63	1						
OM	−0.74 *	−0.63	−0.98 **	1					
CP	0.80 **	0.70 *	0.98 **	−0.98 **	1				
NDF	−0.82 **	−0.72 *	−0.95 **	0.95 **	−0.99 **	1			
ADF	−0.81 **	−0.71 *	−0.96 **	0.96 **	−0.99 **	0.99 **	1		
GE	0.19	0.17	0.27	−0.27	0.26	−0.23	−0.27	1	
DM	−0.69 *	−0.64 *	−0.5	0.5	−0.5	0.54	0.53	−0.33	1

Note: DE, digestible energy; ME, metabolizable energy; Ash, crude ash; OM, organic matter; CP, crude protein; NDF, neutral detergent fiber; ADF, acid detergent fiber; GE, gross energy; DM, dry matter. * *p* < 0.05; ** *p* < 0.01.

**Table 19:** Correlation analysis of routine chemical compositions and SID AAs of FCSM and FCSP fed to growing pigs.

Items	DM	Ash	OM	CP	NDF	ADF	GE	Free Gossypol	SID Thr	SID Lys	SID Trp	SID Met
DM	1											
Ash	−0.5 **	1										
OM	0.09	−0.42 *	1									
CP	−0.47 **	0.98 **	−0.44 **	1								
NDF	0.45 **	−0.95 **	0.52 **	−0.99 **	1							
ADF	0.45 **	−0.96 **	0.5 **	−0.99 **	1 **	1						
GE	−0.63 **	0.29 *	0.13	0.28 *	−0.25	−0.29 *	1					
Free gossypol	0.29 *	−0.93 **	0.37 **	−0.94 **	0.94 **	0.94 **	−0.24	1				
SID Thr	−0.16	−0.17	0.13	−0.24	0.24	0.25	0.11	0.13	1			
SID Lys	−0.05	−0.31 *	0.28 *	−0.39 **	0.4 **	0.4 **	0.1	0.24	0.94 **	1		
SID Trp	−0.24	0.77 **	−0.22	0.81 **	−0.78 **	−0.8 **	0.21	−0.75 **	−0.22	−0.37 *	1	
SID Met	−0.09	0.61 **	−0.12	0.65 **	−0.64 **	−0.64 **	0.2	−0.75 **	0.01	−0.02	0.70 **	1

Note: DM, dry matter; Ash, crude ash; OM, organic matter; CP, crude protein; NDF, neutral detergent fiber; ADF, acid detergent fiber; GE, gross energy; Thr, threonine; Lys, lysine; Trp, tryptophan; Met, methionine. * *p* < 0.05; ** *p* < 0.01.

**Table 20:** Stepwise regression equation of available energy and SID AAs of FCSM and FCSP fed to growing pigs.

NO.	Equation for Fermented Cottonseed Meal/Fermented Cottonseed Protein (*n* = 10)	RMSE	*p* Value	R^2^
1	DE (MJ/kg DM)=20.96−5.52 Ash+1.02 CP+0.56 ADF−0.23 DM	1.50	<0.05	0.81
2	ME (MJ/kg DM)=2.13−0.42 Ash+0.02 DM+0.91 DE	0.71	<0.01	0.93
3	SID Lys (%)=298.32−1.37 DM−4.50 Ash−0.50 OM+1.26 NDF−3.73 GE−0.08 free gossypol	2.66	<0.01	0.71
4	SID Met (%)=−2.1−4.45 Ash−0.05 OM+0.81 CP+0.73 ADF−0.01 free gossypol	0.61	<0.01	0.76
5	SID Thr (%)=156.92−0.77 DM−0.36 OM+0.80 NDF−2.22 GE−0.04 free gossypol	1.99	<0.01	0.57
6	SID Trp (%)=−16.01+0.06 DM−2.25 Ash+0.55 CP+0.20 NDF	0.64	<0.01	0.78

Note: DM, dry matter; Ash, crude ash; OM, organic matter; CP, crude protein; NDF, neutral detergent fiber; ADF, acid detergent fiber; GE, gross energy; Thr, threonine; Lys, lysine; Trp, tryptophan; Met, methionine; SID, standardized ileal digestibility. “*n* = 10” represents the number of fermented cottonseed meal/protein (FCSM/FCSP) samples. The *p* column represents the overall significance test for each regression model.

## Data Availability

The original contributions presented in this study are included in the article/Supplementary Material. Further inquiries can be directed to the corresponding author.

## Supplementary Materials

The following supporting information can be downloaded at https://www.mdpi.com/article/10.3390/ani16213427/s1, Table S1: Fermentation substrate and fermentation parameters. Table S2: A Youden square design in Exp. 2. Table S3: A Youden square design in Exp. 3.

## Abbreviations

The following abbreviations are used in this manuscript:

AA	Amino acid
ADF	Acid detergent fiber
AID	Apparent ileal digestibility
ATTD	Apparent total tract digestibility
BW	Body weight
CP	Crude protein
CV	Coefficient of variation
DE	Digestible energy
DM	Dry matter
FCSP	Fermented cottonseed protein
FCSM	Fermented cottonseed meal
FG	Free gossypol
GE	Gross energy
ME	Metabolizable energy
NDF	Neutral detergent fiber
OM	Organic matter
RMSE	Root mean square error
SEM	Standard error of the mean
SID	Standardized ileal digestibility

## References

[1] Hu Q.L., Shi H.W., Wang L., Wang L., Hou Y., Wang H.L., Lai C.H., Zhang S. (2023). Mitigating environmental impacts using net energy system in feed formulation in China’s pig production. Sci. Total Environ..

[2] Ministry of Agriculture and Rural Affairs of the People’s Republic of China National Pig Industry Statistical Data 2023. https://www.moa.gov.cn/ztzl/szcpxx/jdsj/2023/202312/.

[3] Khan S., Naz S., Sultan A., Alhidary I.A., Abdelrahman M.M., Khan R.U., Khan N.A., Khan M.A., Ahmad S. (2016). Worm meal: A potential source of alternative protein in poultry feed. World’s Poult. Sci. J..

[4] Khan M.A., Wahid A., Ahmad M., Tahir M.T., Ahmed M., Ahmad S., Hasanuzzaman M., Ahmad S., Hasanuzzaman M. (2020). World Cotton Production and Consumption: An Overview. Cotton Production and Uses.

[5] Gao D., Cao Y., Li H. (2010). Antioxidant activity of peptide fractions derived from cottonseed protein hydrolysate. J. Sci. Food Agric..

[6] Song W.G., Kong X.Z., Hua Y.F., Li X.F., Zhang C.M., Chen Y.M. (2020). Antioxidant and antibacterial activity and in vitro digestion stability of cottonseed protein hydrolysates. LWT.

[7] Desrochers P., Szurmak J. (2017). Long distance trade, locational dynamics and by-product development: Insights from the history of the American cottonseed industry. Sustainability.

[8] Tao A., Wang J.H., Luo B., Liu B.W., Wang Z.R., Chen X.P., Zou T.D., Chen J., You J.M. (2024). Research progress on cottonseed meal as a protein source in pig nutrition: An updated review. Anim. Nutr..

[9] Nagalakshmi D., Rama Rao S.V., Panda A., Sastry V.R.B. (2007). Cottonseed meal in poultry diets: A review. J. Poult. Sci..

[10] Mudarra R.A., Tsai T.C.C., Hansen C., Littlejohn B.P., Maxwell C.V., Rorie R. (2021). Effect of gossypol from cottonseed meal on growth performance, plasma gossypol, and complete blood cell counts in commercial growing pigs: A preliminary study on feral hog control. J. Anim. Sci..

[11] Lv L.K., Xiong F.L., Pei S.T., He S.S., Li B.O., Wu L.J., Cao Z.J., Li S.L., Yang H.J. (2025). Synergistic fermentation of cottonseed meal using Lactobacillus mucosae LLK-XR1 and acid protease: Sustainable production of cottonseed peptides and depletion of free gossypol. Food Chem..

[12] Adeola O., Lewis A.J., Southern L.L. (2001). Digestion and balance assays. Swine Nutrition.

[13] National Research Council (2012). Nutrient Requirements of Swine.

[14] Stein H.H., Shipley C.F., Easter R.A. (1998). Technical note: A technique for inserting a T-cannula into the distal ileum of pregnant sows. J. Anim. Sci..

[15] Pan L., Ma X.K., Wang H.L., Xu X., Zeng Z.K., Tian Q.Y., Zhao P.F., Zhang S., Yang Z.Y., Piao X.S. (2016). Enzymatic feather meal as an alternative animal protein source in diets for nursery pigs. Anim. Feed. Sci. Technol..

[16] AOAC International (2012). Official Methods of Analysis.

[17] AOAC International (2016). Official Methods of Analysis.

[18] Van Soest P.J., Robertson J.B., Lewis B.A. (1991). Methods for dietary fiber analysis. J. Dairy Sci..

[19] (2020). Method for Determination of Free Gossypol in Feeds.

[20] AOAC International (2006). Official Methods of Analysis.

[21] (2008). Soy Peptides Powder.

[22] AOCS (2017). Official Method Ba 11-65: Nitrogen solubility index (NSI). Official Methods and Recommended Practices of the AOCS.

[23] (2019). Determination of Amino Acids in Feeds.

[24] Adeola O., Lewis A.J., Southern L.L. (2001). Calculation of ingredient digestibility via substitution method. Swine Nutrition.

[25] Xue L., Zhang C., Cheng B., Song Q., Johnston L.J., Liu L., Wang F.L., Zang J.J. (2025). Prediction equations for net energy of gestating sow soybean meal. Anim. Biosci..

[26] Stein H.H., Kim S.W., Nielsen T.T., Easter R.A. (2001). Standardized ileal digestibility of protein and amino acids in growing pigs and sows. J. Anim. Sci..

[27] Stein H.H., Roth J.A., Sotak K.M., Rojas O.J. Nutritional Value of Soy Products Fed to Pigs. Swine Focus 2013. https://nutrition.ansci.illinois.edu/SwineFocus004.

[28] Kumar M., Tomar M., Punia S., Grasso S., Arrutia F., Choudhary J., Singh S., Verma P., Mahapatra A., Patil S. (2021). Cottonseed: A sustainable contributor to global protein requirements. Trends Food Sci. Technol..

[29] He Z.Q., Zhang H.L., Olk D.C. (2015). Chemical composition of defatted cottonseed and soy meal products. PLoS ONE.

[30] Dai Z.J., Zhang M.S., Li Z.W., Lu D.L., Wu L., Qin C., Wang H.N., Deng J.J., Luo X.C. (2025). Highly efficient and sustainable bioconversion of cottonseed meal to high-value products through solid-state fermentation by protease-enhanced Streptomyces sp. SCUT-3. Chem. Eng. J..

[31] Zhuge Q., Posner E.S., Deyoe C.W. (1988). Production study of a low-gossypol protein product from cottonseed meal. J. Agric. Food Chem..

[32] Ma X.K., Hu J.X., Shang Q.H., Liu H.S., Piao X.S. (2019). Chemical composition, energy content and amino acid digestibility in cottonseed meals fed to growing pigs. J. Appl. Anim. Res..

[33] Mageshwaran V., Satankar V., Paul S. (2023). Solid-state fermentation for gossypol detoxification and nutritive enrichment of cottonseed cake: A scale-up of batch fermentation process. BioResources.

[34] Li J., Gao T.G., Hao Z.M., Guo X.J., Zhu B.C. (2022). Anaerobic solid-state fermentation with Bacillus subtilis for digesting free gossypol and improving nutritional quality in cottonseed meal. Front. Nutr..

[35] Tropea A., Ferracane A., Albergamo A., Potortì A.G., Lo Turco V., Di Bella G. (2022). Single cell protein production through multi food-waste substrate fermentation. Fermentation.

[36] Lin J.C., Zhang J.X., Zou G., Zhang X.L., Shang H.H., Ji B.Y., Bai Y.Y., Qu L.B., Wei Y.J. (2024). Enhancing the nutritional quality of defatted cottonseed meal by solid-state fermentation with probiotic microbes. Fermentation.

[37] Choi K.Y., Wernick D.G., Tat C.A., Liao J.C. (2014). Consolidated conversion of protein waste into biofuels and ammonia using Bacillus subtilis. Metab. Eng..

[38] Ni S., Zhao D., Li K., Wu Y., Yang S., Chen X., Cui Z., Yan X., Liu G. (2024). Cofermentation of Cottonseed Meal by the Synergistic Action of Microbial Flora with Protease and Its Metabolic Kinetic. ACS Food Sci. Technol..

[39] Lopez H.W., Krespine V., Guy C., Messager A., Demigne C., Remesy C. (2001). Prolonged fermentation of whole wheat sourdough reduces phytate level and increases soluble magnesium. J. Agric. Food Chem..

[40] Rodríguez Muela C., Rodríguez Ramírez H.E., Díaz Plascencia D., Bocourt Salabarria R., Arzola Álvarez C. (2017). Concentration of acid detergent fiber, neutral detergent fiber and lignin during solid state fermentation of apple (Malus domestica) derivates. Cuban J. Agric. Sci..

[41] Yadav A., Ali A.M., Ingawale M., Raychaudhuri S.S., Gantayet L.M., Pandit A.B. (2020). Enhanced co-production of pectinase, cellulase and xylanase enzymes from Bacillus subtilis ABDR01 upon ultrasonic irradiation. Process Biochem..

[42] Ren F.Y., Wu F., Jie Y.C., Wu X., Gao L. (2025). Carbon-nitrogen metabolic coupling for optimized protein production of corn stover polysaccharides: From molecular mechanisms to buffalo rumen microbiome. Int. J. Biol. Macromol..

[43] Liu Y., Luan J., Feng X., Zhou J., Geng C. (2026). Evaluation of the feeding value of microbially fermented cottonseed meal by in vitro method. Czech J. Anim. Sci..

[44] Esen S. (2024). Effect of solid-state fermented cottonseed meal on broiler growth performance, carcass traits and blood biochemical parameters: A systematic review. Fermentation.

[45] Villamide M.J. (1996). Methods of energy evaluation of feed ingredients for rabbits and their accuracy. Anim. Feed. Sci. Technol..

[46] Feng D.Y. (2003). Formulation Feed.

[47] Hu Q., Wang F.L., Piao X.S., Ni J.J., Zhang X.M., Li D.F. (2012). Effects of Fasting Duration and Body Weight on Fasting Heat Production in Growing Pigs. J. Anim. Vet. Adv..

[48] Yue Y.R., Lin J.W., Lv G., Liu B.C., Deng X.Y., Li Y.J., Li X.B., Chen K.X. (2025). Effects of replacing soybean meal with cottonseed meal in amino acid balanced diets on growth performance, apparent digestibility, ruminal fermentation, and microbial diversity in fattening Dorper × Hu crossbred sheep. Front. Vet. Sci..

[49] Gu X.L., Li Z.Q., Wang J., Chen J.S., Jiang Q., Liu N., Liu X., Zhang F., Tan B., Li H. (2021). Fermented cottonseed meal as a partial replacement for soybean meal could improve the growth performance, immunity and antioxidant properties, and nutrient digestibility by altering the gut microbiota profile of weaned piglets. Front. Microbiol..

[50] Ainiwaer Z., Yang Z.J., Luo Q.Y., Xu T.X., Wang C.D. (2026). Effects of protease-hydrolyzed cottonseed protein on growth performance, intestinal morphology, and bacterial diversity in broilers. Poult. Sci..

[51] Zhang W.J., Xu Z.R., Sun J.Y., Yang X. (2006). Effect of selected fungi on the reduction of gossypol levels and nutritional value during solid substrate fermentation of cottonseed meal. J. Zhejiang Univ. Sci. B.

[52] de Castro R.J.S., Nishide T.G., Sato H.H. (2014). Production and biochemical properties of proteases secreted by Aspergillus niger under solid state fermentation in response to different agroindustrial substrates. Biocatal. Agric. Biotechnol..

[53] Grundy M.M.L., Labarre J., Mayeur-Nickel F., van Milgen J., Renaudeau D. (2023). An in vitro and in vivo approach to characterize digesta from pigs fed different forms of pea flour. J. Anim. Sci..

[54] Su F., Yu B., Sun J.B., Ou H.Y., Zhao B., Wang L.M., Qin J.Y., Tang H., Tao F., Jarek M. (2011). Genome sequence of the thermophilic strain Bacillus coagulans 2-6, an efficient producer of high-optical-purity L-lactic acid. J. Bacteriol..

[55] Georgieva R., Iliev I., Haertlé T., Chobert J.M., Ivanova I., Danova S. (2009). Technological properties of candidate probiotic Lactobacillus plantarum strains. Int. Dairy J..

[56] Bueno-Mancebo J., Artola A., Barrena R., Sánchez A., Gea T. (2026). Emerging solid-state fermentation in functional foods: Bioactive compounds, functionality, sensory quality, microbiota influence and industrial perspectives. Fermentation.

[57] Boisen S., Fernández J.A. (1995). Prediction of the apparent ileal digestibility of protein and amino acids in feedstuffs and feed mixtures for pigs by in vitro analyses. Anim. Feed. Sci. Technol..

[58] Liu K.L., Zheng J.B., Chen F.S. (2021). Heat-induced changes in the physicochemical properties and in vitro digestibility of rice protein fractions. J. Food Sci. Technol..

[59] Wisbech S.J., Jørgensen H., Nielsen T.S., Bach Knudsen K.E. (2023). Influence of fiber-rich coproducts on nutrient and energy digestibility and utilization in sows. J. Anim. Sci..

[60] Yoshimura E., Oi N., Abe K., Nishida Y. (2026). Digestible and metabolizable energy intake in humans: A systematic review. Adv. Nutr..

[61] Wang J.F., Zhu Y.H., Li D.F., Jørgensen H., Jensen B.B. (2004). The influence of different fiber and starch types on nutrient balance and energy metabolism in growing pigs. Asian-Australas. J. Anim. Sci..

[62] Aiello S.E., Moses M.A. (2016). The Merck Veterinary Manual.

