# Supplementary material for: Nutrient and Amino Acid Digestibility of Fermented Cottonseed Meal and Fermented Cottonseed Protein Produced with Different Microbial Combinations in Growing Pigs
Source: Animals (Basel). 2026 Oct 30;16(21):3427. doi: 10.3390/ani16213427 (PMC13761581; doi:10.3390/ani16213427)
Supplement: Supplementary file 1 [file animals-16-03427-s001.zip › animals-4533708-supplementary.pdf]

Table S1. Fermentation substrate and fermentation parameters.

Sample ID	Strain/Composition	Time Point (C1-C5)	Condition
1	B	C1	Aerobic
2	B	C2	Aerobic
3	B	C3	Aerobic
4	B	C4	Aerobic
5	B+G	C5	Aerobic
6	A	C1	Aerobic
7	A	C2	Aerobic
8	A	C3	Aerobic
9	A+G	C4	Aerobic
10	A	C5	Aerobic
11	D	C1	Aerobic
12	D	C2	Aerobic
13	D	C3	Aerobic
14	D	C4	Aerobic
15	D	C5	Aerobic
16	C	C1	Aerobic
17	C	C2	Aerobic
18	C	C3	Aerobic
19	C	C4	Aerobic
20	C	C5	Aerobic
21	C	C1	Anaerobic
22	F	C1	Anaerobic
23	D	C1	Anaerobic
24	E	C1	Anaerobic
25	D	C3	Anaerobic
26	E	C3	Anaerobic
27	F	C3	Anaerobic
28	C	C3	Anaerobic
29	D+C	C1	Anaerobic
30	F+C	C1	Anaerobic
31	E+C	C1	Anaerobic
32	D+F	C1	Anaerobic
33	E+F	C1	Anaerobic
34	D+E	C1	Anaerobic
35	D+C	C3	Anaerobic
36	F+C	C3	Anaerobic
37	E+C	C3	Anaerobic
38	D+F	C3	Anaerobic
39	E+F	C3	Anaerobic
40	D+E	C3	Anaerobic
41	D+C+G	C1	Anaerobic
42	F+C+G	C1	Anaerobic

43	E+C+G	C1	Anaerobic
44	D+F+G	C1	Anaerobic
45	E+F+G	C1	Anaerobic
46	D+E+G	C1	Anaerobic
47	D+C+G	C3	Anaerobic
48	F+C+G	C3	Anaerobic
49	E+C+G	C3	Anaerobic
50	D+F+G	C3	Anaerobic
51	E+F+G	C3	Anaerobic
52	D+E+G	C3	Anaerobic
53	D+E+G	C2	Anaerobic
54	E+C+G	C2	Anaerobic
55	F+C+G	C2	Anaerobic
56	E+F+G	C2	Anaerobic
57	D+F+G	C2	Anaerobic
58	D+C+G	C2	Anaerobic
59	D+E	C2	Anaerobic
60	E+C	C2	Anaerobic
61	F+C	C2	Anaerobic
62	E+F	C2	Anaerobic
63	D+F	C2	Anaerobic
64	D+C	C2	Anaerobic
65	D+E	C4	Anaerobic
66	F+C	C4	Anaerobic
67	E+C	C4	Anaerobic
68	E+F	C4	Anaerobic
69	D+F	C4	Anaerobic
70	D+C	C4	Anaerobic
71	E+F	C5	Anaerobic
72	E+C	C5	Anaerobic
73	D+C	C5	Anaerobic
74	F+C	C5	Anaerobic
75	D+F	C5	Anaerobic
76	D+E	C5	Anaerobic
77	D+E+G	C4	Anaerobic
78	D+C+G	C4	Anaerobic
79	D+F+G	C4	Anaerobic
80	E+F+G	C4	Anaerobic
81	E+C+G	C4	Anaerobic
82	F+C+G	C4	Anaerobic
83	D+E+G	C5	Anaerobic
84	F+C+G	C5	Anaerobic
85	E+F+G	C5	Anaerobic
86	D+F+G	C5	Anaerobic

87	D+C+G	C5	Anaerobic
88	E+C+G	C5	Anaerobic
89	D+C+E	C2	Anaerobic
90	E+C+F	C2	Anaerobic
91	D+E+F	C2	Anaerobic
92	D+C+F	C2	Anaerobic
93	E+F+C+D	C2	Anaerobic
94	D+C+E	C5	Anaerobic
95	E+C+F	C5	Anaerobic
96	D+E+F	C5	Anaerobic
97	D+C+F	C5	Anaerobic
98	E+F+C+D	C5	Anaerobic
99	D+C+E+G	C5	Anaerobic
100	E+C+F+G	C5	Anaerobic
101	D+E+F+G	C5	Anaerobic
102	D+C+F+G	C5	Anaerobic
103	E+F+C+D+G	C5	Anaerobic

Note: Aspergillus oryzae (A); Aspergillus niger (B); Saccharomyces cerevisiae (C); Bacillus subtilis (D); Bacillus coagulans (E); Lactobacillus plantarum (F); Neutral protease (G).

Table S2. A Youden square design in Exp. 2.

Pig No.	Period 1	Period 2	Period 3	Period 4	Period 5	Period 6
1	D0	D1	D2	D3	D4	D5
2	D1	D2	D3	D4	D5	D6
3	D2	D3	D4	D5	D6	D7
4	D3	D4	D5	D6	D7	D8
5	D4	D5	D6	D7	D8	D9
6	D5	D6	D7	D8	D9	D10
7	D6	D7	D8	D9	D10	D0
8	D7	D8	D9	D10	D0	D1
9	D8	D9	D10	D0	D1	D2
10	D9	D10	D0	D1	D2	D3
11	D10	D0	D1	D2	D3	D4

Note: D0-D10 represent 11 different diets in Exp. 2.

Table S3. A Youden square design in Exp. 3.

Pig No.	Period 1	Period 2	Period 3
1	D0	D1	D2
2	D1	D2	D3
3	D2	D3	D4
4	D3	D4	D5
5	D4	D5	D6
6	D5	D6	D7

7	D6	D7	D8
8	D7	D8	D9
9	D8	D9	D10
10	D9	D10	D0
11	D10	D0	D1
12	D0	D1	D2
13	D1	D2	D3
14	D2	D3	D4
15	D3	D4	D5
16	D4	D5	D6
17	D5	D6	D7
18	D6	D7	D8
19	D7	D8	D9
20	D8	D9	D10
21	D9	D10	D0
22	D10	D0	D1

Note: D0-D10 represent 11 different diets in Exp. 3.
